# Arc-Length Re-Parametrization and Signal Registration to Determine a Characteristic Average and Statistical Response Corridors of Biomechanical Data

**DOI:** 10.3389/fbioe.2022.843148

**Published:** 2022-03-24

**Authors:** Devon C. Hartlen, Duane S. Cronin

**Affiliations:** Department of Mechanical Engineering, University of Waterloo, Waterloo, ON, Canada

**Keywords:** arc-length re-parameterization, biomechanical data, statistical response corridors, characteristic average curve, biofidelity, force-displacement, acceleration-time

## Abstract

A characteristic average and biofidelity response corridors are commonly used to represent the average behaviour and variability of biomechanical signal data for analysis and comparison to surrogates such as anthropometric test devices and computational models. However, existing methods for computing the characteristic average and corresponding response corridors of experimental data are often customized to specific types or shapes of signal and therefore limited in general applicability. In addition, simple methods such as point-wise averaging can distort or misrepresent important features if signals are not well aligned and highly correlated. In this study, an improved method of computing the characteristic average and response corridors of a set of experimental signals is presented based on arc-length re-parameterization and signal registration. The proposed arc-length corridor method was applied to three literature datasets demonstrating a range of characteristics common to biomechanical data, such as monotonic increasing force-displacement responses with variability, oscillatory acceleration-time signals, and hysteretic load-unload data. The proposed method addresses two challenges in assessing experimental data: arc-length re-parameterization enables the assessment of complex-shaped signals, including hysteretic load-unload data, while signal registration aligned signal features such as peaks and valleys to prevent distortion when determining the characteristic average response. The arc-length corridor method was shown to compute the characteristic average and response corridors for a wide range of biomechanical data, while providing a consistent statistical framework to characterize variability in the data. The arc-length corridor method is provided to the community in the freely available and open-source software package, ARCGen.

## Introduction

Experimental data collected from biological tissues, volunteer studies, or post-mortem human subject testing is used to assess or validate surrogates, such as computational models and anthropomorphic test devices, but is often highly variable (Schmitt et al., 2014). This variability complicates the assessment and validation of biomechanical surrogates (Yoganandan et al., 2015), where an average response and statistical representation of variability are needed. Quantitative curve matching assessments such as r-squared, root mean square error, or more sophisticated techniques like dynamic time warping require a single representative or average curve to compare experimental data to surrogate models (Kim et al., 2013). In contrast, biofidelity response corridors have long been used to represent the variability of experimental data (e.g., CORA (Gehre et al., 2009)). However, the fundamental challenge of computing an average curve and corridors remains ill-defined and is often tailored to specific applications such that existing methods are not generally applicable.

Documented methods of computing a characteristic average and biomechanical response corridors have been broadly divided into time-based and cross-variable (representing one measured variable with respect to a second measured variable, such as force-displacement) methods (Kim et al., 2013). While all signals are, to one extent or another, time-based, the time component of a signal may not be applicable or useful, such as in the case of quasi-static mechanical characterization testing or in cases where time information may not be available, such as reproducing force-displacement responses from publications.

Due to the prevalence of time-series data in experimental work, time-based methods are among the most mature and widely used to determine characteristic curves from experimental data (Barker and Cronin, 2021). Time-based methods have generally relied on point-wise calculation of average values and statistics such as a point-wise standard deviation (Kim et al., 2013), requiring all experimental signals to be collected at a uniform rate and to have the same number of points. Unequally space data or signals of different sampling rates can be incorporated but must be resampled to match the sampling rate and the number of data points as the other signals being analyzed.

However, a challenge for time-based methods is that point-wise calculations can misrepresent or distort characteristics of the signals if critical features such as peaks and valleys are not aligned in time. Such misalignment could result from physical variation between individual experimental tests, or experimental difficulties such as synchronizing the start of time-series data between different tests. A range of methods have been presented to align signals based on techniques such as alignment of a characteristic time (Cavanaugh et al., 1986; Maltese et al., 2002) and alignment to minimize variation (Morgan et al., 1981; Eppinger et al., 1984) or maximized correlation between signals (Nusholtz et al., 2009; Donnelly et al., 2017), generally by shifting signals in time relative to one another. These phase-shift techniques have seen success in minimizing distortion during averaging but can become less effective if signals contain more than one critical feature (Ramsay, 2018).

Signal registration (also called curve registration) is a technique borrowed from the field of functional data analysis (Ramsay and Li, 1998; Tang and Muller, 2008; Gasser and Kneip, 2012; Ramsay, 2018) for aligning multiple features of time-series data simultaneously to compute a characteristic average. The principal behind signal registration is the introduction of warping functions, $h_{i}$ , that non-linearly scale portions of each signal to align features such as peaks and valleys between all signals without altering the underlying shape of each signal. Warping functions are determined iteratively by optimizing for a range of metrics such as cross-correlation or mean squared error between all curves. As opposed to the phase-shift methods discussed earlier, signal registration provides the ability to align many shared features simultaneously, helping to minimize distortion and better capture the underlying shape of the data (Ramsay and Li, 1998; Ramsay, 2018).

Computing corridors for cross-variable signals presents a number of challenges. While researchers have successfully combined the average and standard deviation of two time-series signals to produce cross-variable response corridors (Cavanaugh et al., 1986), this method is only effective when signals are highly correlated and when the original sampling time data is available. Further, point-wise averages of signals based on one of the cross-variable signal axes can result in loss of the characteristic shape of the underlying data (Kim et al., 2013; Mattucci and Cronin, 2015) or entirely fail if signals do not extend to the same point on each axis (Lessley et al., 2004; Mattucci and Cronin, 2015), such as differences in displacement to failure.

A historical method of generating corridors for cross-variable data is the so-called ‘eyeball average’, wherein a characteristic average and corridors are drawn based on how the researcher interprets the signals (Lobdell et al., 1972). Unfortunately, this methodology is highly subjective and does not characterize the average curve or statistical variability of the data. The work of Lessley et al. (2004) presents one of the first rigorous methods for computing a characteristic average and corridors by normalizing each cross-variable signal against itself, then computing the average and standard deviation for each point in the normalized curves Shaw et al. (2006) later implemented an improved statistical representation of signals based on a two-dimensional normal distribution. However, both the methods were limited to monotonic data, requiring non-monotonic signals to be divided into strictly monotonic segments. Consistent segmentation can be challenging for highly variable signals or signals without a well-defined characteristic shape Perez-Rapela et al. (2018) eliminated the need for segmentation by re-parameterizing signals based on their arc-length, rather than ordinate and abscissa values. Perez-Rapela further utilized a simple method for aligning signal features by normalizing arc-length calculations to a feature shared between signals, such as a characteristic peak that appears in every signal.

While biomechanical data takes the forms of time-based or cross-variable signals, the overall shape and characteristics of the signals are equally important metrics as these factors place limitations on the methods used to analyze the signals. To that end, biomechanical data can be broadly divided into three categories based on signal shape. The first category includes strictly monotonic signals, such as the mechanical response of biological tissues such as ligament force-displacement signals (Mattucci et al., 2012; Mattucci and Cronin, 2015). Second, signals that exhibit oscillatory or otherwise non-monotonic behaviour in one axis. Acceleration-time signals, such as head acceleration under rapid deceleration being a classic example (Ewing and Thomas, 1972). Third, signals that exhibit hysteresis or non-monotonic behavior in both axes. The load-unload force-displacement response from impact testing, such pendulum impacts on the thorax of post-mortem human subjects (Kroell et al., 1971), fall under this category.

In the present study, an improved general methodology for computing a characteristic average and statistical response corridors from biomechanical data is presented to address many of the limitations encountered in previously documented methods. The proposed method incorporates arc-length re-parameterization and signal registration to enable general applicability, and was applied to three distinct signal types: monotonic increasing data (force-displacement) with variability, oscillatory acceleration-time data, and hysteretic load-unload force-displacement data.

## Methodology

**FIGURE 1:**
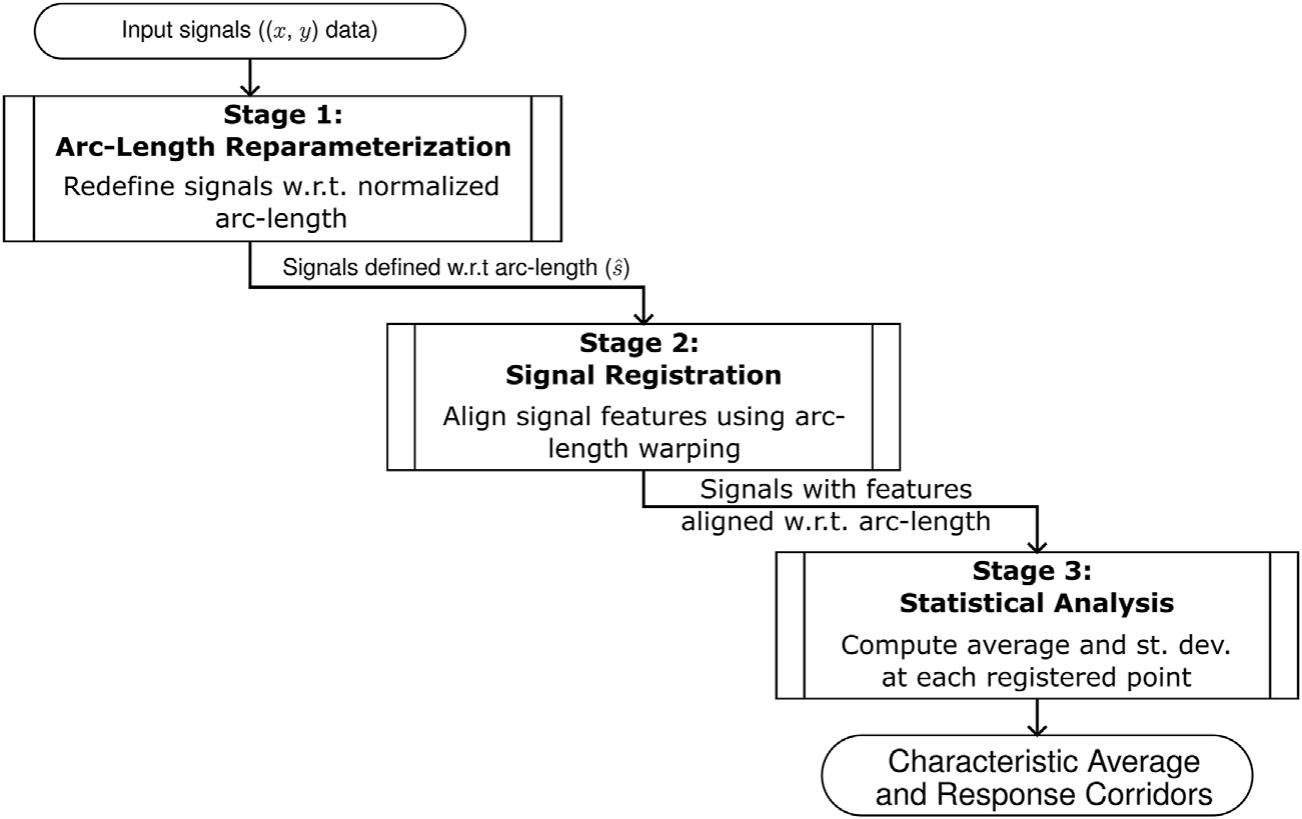
An overview of the three stages of the arc-length corridor method used to compute the characteristic average and response corridors of a set of inputs signals.

The proposed method of computing a characteristic average and response corridor outputs from a set of input signals presented here can be divided into three stages: arc-length re-parameterization, signal registration, and statistical analysis (Figure 1). The analysis considers a set of n signals, $y_{i}\left(x\right)$ ( i=1,…,n ) where the j ^th^ point of the i ^th^ signal is defined as $y_{i,j}$ . It should be noted that the input signals do not need to contain the same number of data points, nor is a constant sampling rate required, as resampling was undertaken during the re-parameterization process.

### Stage 1: Arc-Length Re-Parameterization of Input Signals

**FIGURE 2:**
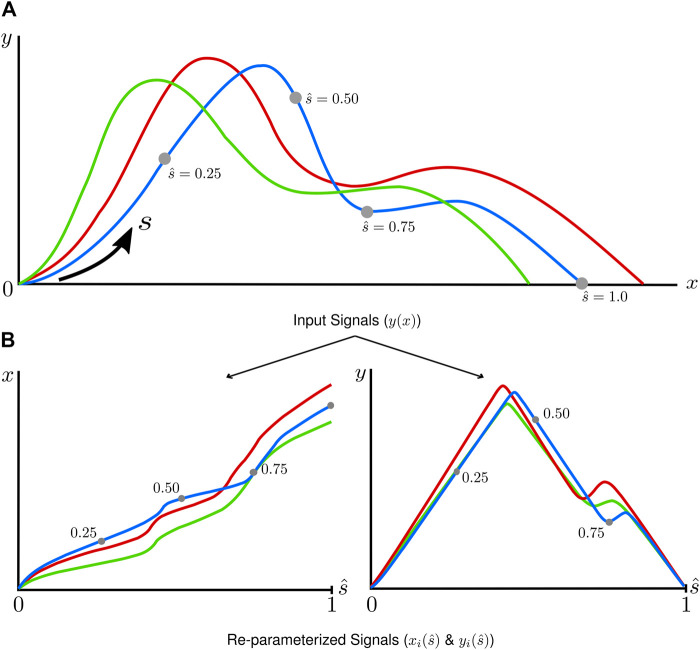
A graphical depiction of the arc-length re-parameterization process, where input signals **(A)** defined y(x) are re-parameterized into two curves ( $x_{i}\left(\hat{s}\right)$ and $y_{i}\left(\hat{s}\right)$ ) with respect to arc length **(B)**.

In the first stage, individual signals were re-parametrized with respect to arc-length, a monotonically increasing metric intrinsically tied to the shape of each signal (Figure 2). Each signal was first scaled based on the total range of the data (Eqs 1, 2) such that the ordinate and abscissa values were of the same order in magnitude.

$${\hat{x}}_{i,j}=\frac{x_{i,j}}{{\bar{x}}_{max}-{\bar{x}}_{min}}$$ (1)

$${\hat{y}}_{i,j}=\frac{y_{i,j}}{{\bar{y}}_{max}-{\bar{y}}_{min}}\;\;$$ (2)

where

$${\bar{x}}_{min}=\frac{1}{n}\overset{n}{\underset{i=1}{\sum }}\min\left(x_{i}\right)$$ (3)

$${\bar{x}}_{max}=\frac{1}{n}\overset{n}{\underset{i=1}{\sum }}\max\left(x_{i}\right)$$ (4)

$${\bar{y}}_{min}=\frac{1}{n}\overset{n}{\underset{i=1}{\sum }}\min\left(y_{i}\right)$$ (5)

$${\bar{y}}_{max}=\frac{1}{n}\overset{n}{\underset{i=1}{\sum }}\max\left(y_{i}\right)$$ (6)

In the scaling equations, ${\hat{x}}_{i}$ and ${\hat{y}}_{i}$ are the scaled components of each signal. By scaling signals by the mean extrema of all signals, the relative shape and size of each signal relative to one another are maintained. It is important to note that ${\hat{x}}_{i}$ and ${\hat{y}}_{i}$ are only used for arc-length calculations. Unscaled signal components $x_{i}$ and $y_{i}$ are used exclusively in later signal registration and statistical analysis stages.

Following scaling, the arc-length of each j ^th^ data point ( $s_{i,j}$ ) within each individual i ^th^ signal was computed with zero arc length start corresponding to the first point of the signal. Linear behavior was assumed between discrete points of the signal to compute arc-length for simplicity and was found to be reasonable for the data sets considered in this study (Eq. 7). Higher-order interpolations can be applied in cases where the data is sparse. The arc-length corresponding to each point in the signal was then normalized using the total arc-length of each signal, where ${\hat{s}}_{i}$ denotes normalized arc-length and the total arc-length is equal to the arc-length at the final data point. At this point, both axes of each signal, both scaled and unscaled, are defined with respect to normalized arc-length, such that $x_{i}\left({\hat{s}}_{i}\right)$ and $y_{i}\left({\hat{s}}_{i}\right)$ and the normalized arc-length of each signal ${\hat{s}}_{i}$ ranges from 0 to 1. Finally, all signals were resampled at regular intervals with respect to normalized arc-length $\hat{s}$ , ensuring all signals had the same number of points at the same normalized arc-lengths. This step is necessary as varying slopes in regularly sampled data will result in irregular arc-lengths between discrete signal points. A uniform arc-length between each point is necessary for statistical calculations in stage 3.

$$s_{i,j}=\;\overset{j}{\underset{k=2}{\sum }}\sqrt{{\left(x_{i,k}-x_{i,k-1}\right)}^{2}+{\left(y_{i,k}-y_{i,k-1}\right)}^{2}}$$ (7)

### Stage 2: Signal Registration to Align Critical Features

**FIGURE 3:**
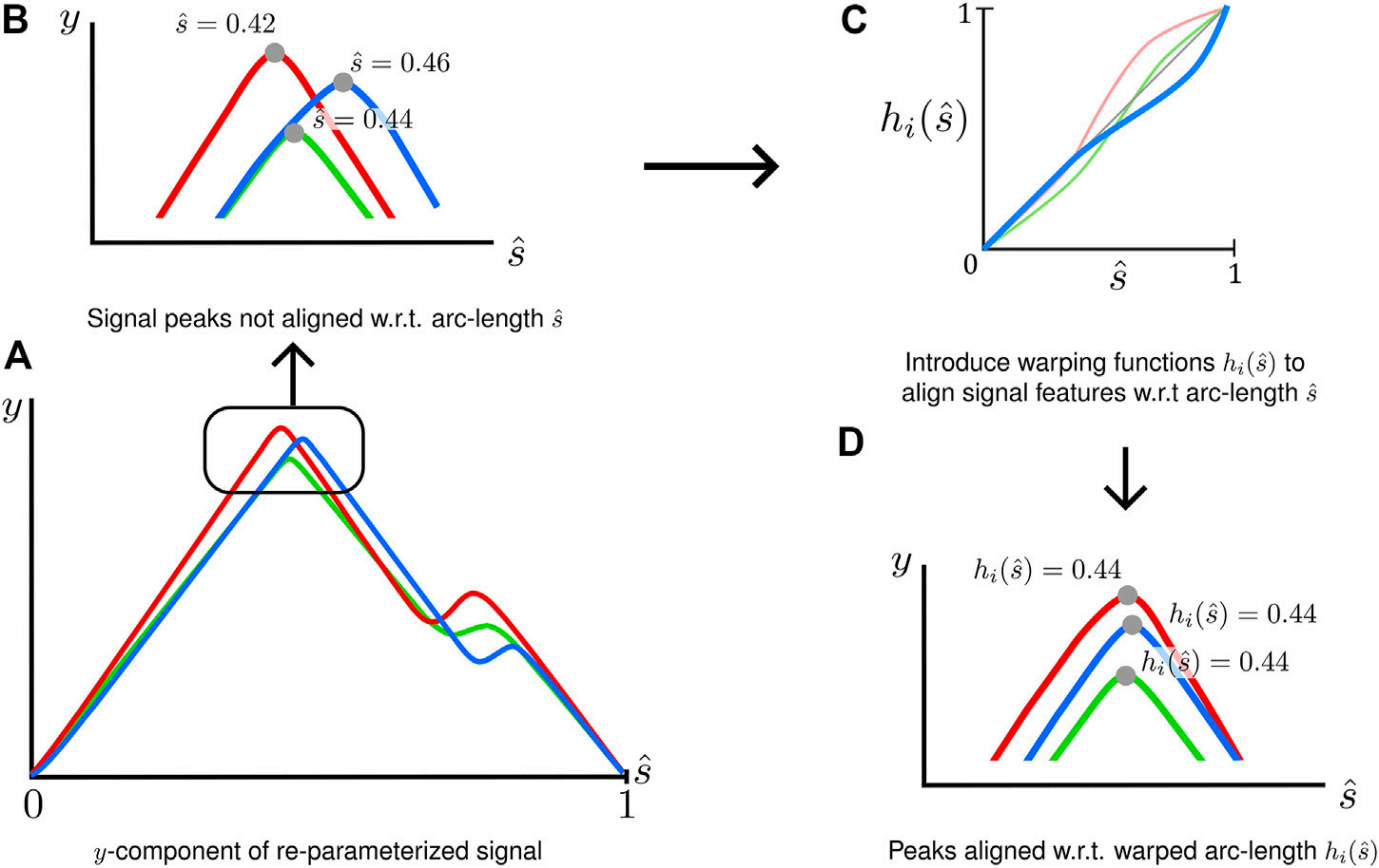
A graphical depiction of the signal registration process to align critical features **(A)** Signals in the arc-length space may not have key features aligned. **(B)** Peaks occurring a different normalized arc-lengths **(C)** Warping functions are introduced for each signal $h_{i}\left(\hat{s}\right)$ that remap the normalized arc-length of each signal **(D)** to align features. Signal registration operates on arc-length, not the underlying (x,y) data of the signal.

Signal registration was applied to align features of all n signals simultaneously (Figure 3) and address an embedded assumption in arc-length re-parameterization, that critical features occur at approximately the same normalized arc-length across all signals.

Signal registration used of strictly monotonic, signal-specific warping functions, $h_{i}\left(\hat{s}\right)$ , to continuously align features across all signals simultaneously by remapping normalized arc-length. Warping functions $h_{i}\left(\hat{s}\right)$ were chosen to be monotonic cubic Hermite interpolating splines, with exterior control points defined at [0,0] and [1,1]. Signal registration included two user-defined parameters: the number of interior control points, m, used in the warping function, and the penalty factor, λ , used to control the degree of warping. Appropriate selection of these two parameters was important to align signal features while maintaining the shape of the input signals.

**FIGURE 4:**
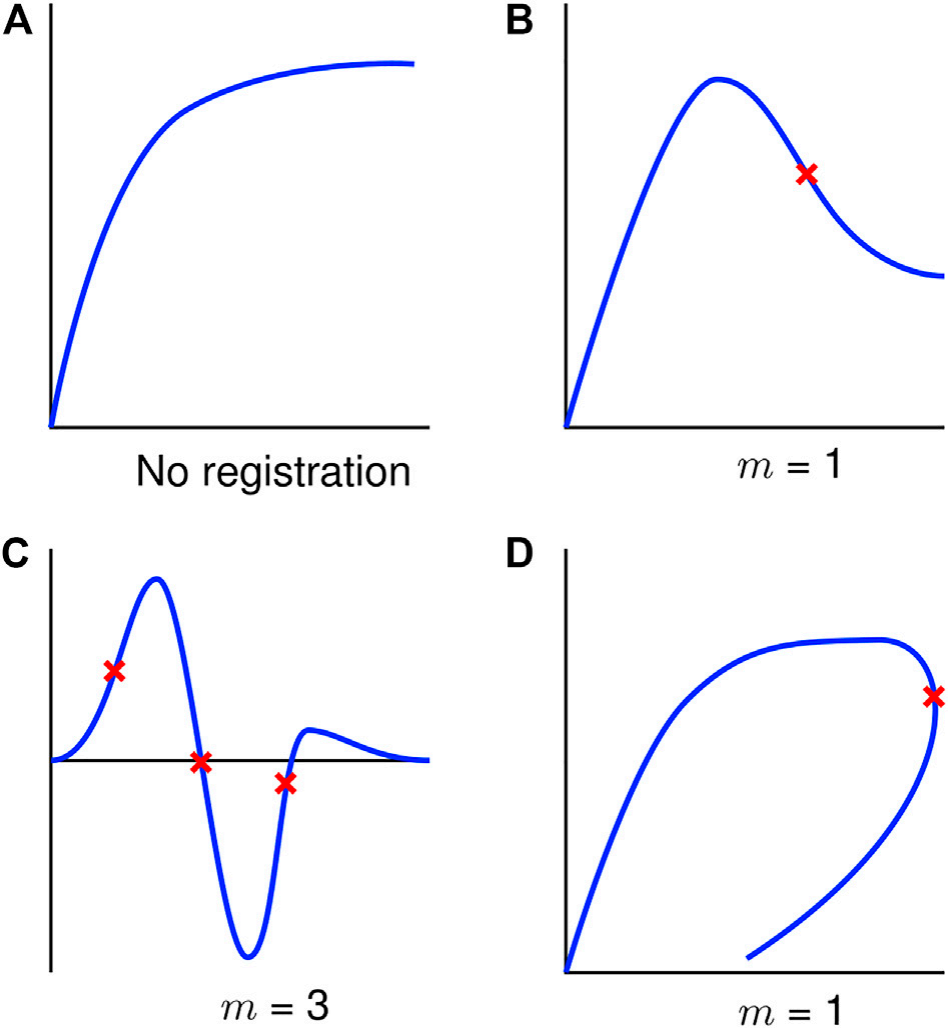
Exemplar signals to aid in selecting the number of control points ( m ) with prominent inflection points denoted with red crosses **(A)** Monotonic signal, no warping needed. **(B)** Non-monotonic, a single control point is used in this signal due to the single inflection point **(C)** Oscillatory signal, three control points corresponding to three inflection points. **(D)** Load-unload signal, one warping point is required for a single inflection point in x versus arc-length response.

The number of interior control points, m, required for a signal was problem-specific; however, some guidance was developed in the current study. It was found that signal registration was generally not required for monotonic data (Figure 4A). However, for non-monotonic data, the number of interior control points should be equal to the number of prominent inflection points present in the characteristic shape of the signal. This number should not include inflection points resulting from small-scale, high-frequency oscillations caused by noise or experimental variation (Figures 4B, C). In addition, some signals may demonstrate load-unload response requiring control points (Figure 4D) corresponding to inflection points in the arc-length space.

The location of the m control points in arc length, for all n signals were determined simultaneously by maximizing the cross-correlation score, C Eq. 8, between all n signals in the arc-length space (Nusholtz et al., 2009), where $c_{ab}$ is the cross-correlation Eq. 9 between two re-parameterized signals in the arc-length space (such as $x_{a}(h_{a}\left(\hat{s}\right))$ and $x_{b}(h_{b}\left(\hat{s}\right))$ or $y_{a}(h_{a}\left(\hat{s}\right))$ and $y_{b}(h_{b}\left(\hat{s}\right))$ ).

$$C=\frac{1}{n\left(n-1\right)}\left(\left(\overset{n}{\underset{i=1}{\sum }}\overset{n}{\underset{j=1}{\sum }}c_{ij}\right)-n\right)$$ (8)

$$c_{ab}=\frac{n\sum_{i}x_{a,i}x_{b,i}-n\;{\bar{x}}_{a,i}\;{\bar{x}}_{b,i}}{\sqrt{\sum_{i}x_{a,i}^{2}-n{{\bar{x}}_{a,i}}^{2}}\sqrt{\sum_{i}x_{b,i}^{2}-n{{\bar{x}}_{b,i}}^{2}}}\;\;\;$$ (9)

As C approaches unity, the cross-correlation between all signals improves. Correlation scores were computed for $x_{i}(h_{i}\left(\hat{s}\right))$ and $y_{i}(h_{i}\left(\hat{s}\right))$ separately, with both values being averaged to form a single optimization score.

In order to prevent shape distortion, warping was limited by a penalty function, Λ Eq. 10, with a defined penalty factor, λ . The penalty function increased as the distance between the warping function $h_{i}\left(\hat{s}\right)$ and unwarped normalized arc-length, $\hat{s}$ , increased.

$$\text{Λ}=\frac{\lambda }{n}\overset{n}{\underset{i=1}{\sum }}\left(\overset{1}{\underset{0}{\int }}\left(h\left(\hat{s}\right)-\hat{s}\right)d\hat{s}\right)$$ (10)

For the penalty factor λ , larger values will minimize the amount of warping introduced during signal registration. A value of $10^{-2}$ was found to align signal features well without compromising the shape or sampling point density of the signals in this work. However, this parameter may need to be changed for other scenarios.

### Stage 3: Statistical Analysis of Re-Parametrized and Registered Signals

After arc-length re-parameterization and signal registration to ensure all signals have the same number of points and have critical features aligned, point-wise statistical analysis was conducted using normalized arc-length. This statistical analysis assumed all (x,y) points at a given normalized arc-length were uncorrelated and normally distributed.

**FIGURE 5:**
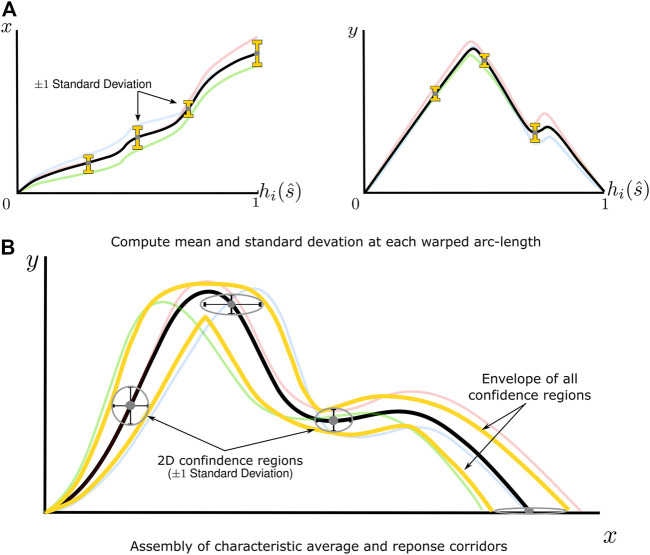
A graphical depiction of the statistical analysis of re-parameterized signals and construction of response corridors **(A)** The mean and standard deviation of signals are computed point-wise in the arc-length space for x and y before being combined. **(B)** Mean x and y values make up the characteristic average, while standard deviations form elliptical confidence regions. The response corridor is defined as the envelope of all elliptical regions.

The characteristic average of the signals was constructed by computing the mean of all (x,y) points at each normalized arc-length (Figure 5). A two-dimensional, uncorrelated normal distribution Eq. 11 was used to compute uncertainty, where $\bar{x}$ and $\bar{y}$ were the mean of all points at a given arc-length, and SD(x) and SD(y) were the standard deviation.

$$f\left(x,y\right)={\left(2\pi \text{SD}\left(x\right)\text{SD}\left(y\right)\right)}^{-1}\exp\left(-\frac{1}{2}\left[{\left(\frac{x-\bar{x}}{\text{SD}\left(x\right)}\right)}^{2}+{\left(\frac{y-\bar{y}}{\text{SD}\left(y\right)}\right)}^{2}\right]\right)$$ (11)

From this distribution, a two-dimensional confidence region can be defined as

$$\chi_{2}^{2}\left(p\right)\geq \;{\left(\frac{x-\bar{x}}{\text{SD}\left(x\right)}\right)}^{2}+{\left(\frac{y-\bar{y}}{\text{SD}\left(y\right)}\right)}^{2}$$ (12)

where $\chi_{2}^{2}\left(p\right)$ is the quantile function (or inverse cumulative density function) of the chi-squared distribution with two degrees of freedom (corresponding to x and y). This confidence region defines an ellipse centered at $\left(\bar{x},\bar{y}\right)$ with axes of $\sqrt{\chi_{2}^{2}\left(p\right)}\text{SD}\left(x\right)$ and $\sqrt{\chi_{2}^{2}\left(p\right)}\text{SD}\left(y\right)$ . One benefit of this definition is that the size of the ellipse can be defined with respect to probability, p . While corridors of plus and minus one standard deviation are common in the literature ( $\chi_{2}^{2}\left(p\right)=1$ ), a confidence interval of this size only encompasses approximately 39.4% ( p = 0.394) of variation. Using the chi-squared distribution makes it possible to define corridors that encompass any amount of variation desired.

Response corridors were defined as the envelope of all elliptical confidence regions at each resampled arc-length value. As determining the envelope of a set of ellipses does not necessarily have a closed-form analytical solution, a marching squares algorithm (Lorensen and Cline, 1987) was used to extract this envelope numerically.

### Evaluation of Three Experimental Datasets Using the Arc-Length Corridor Method

**FIGURE 6:**
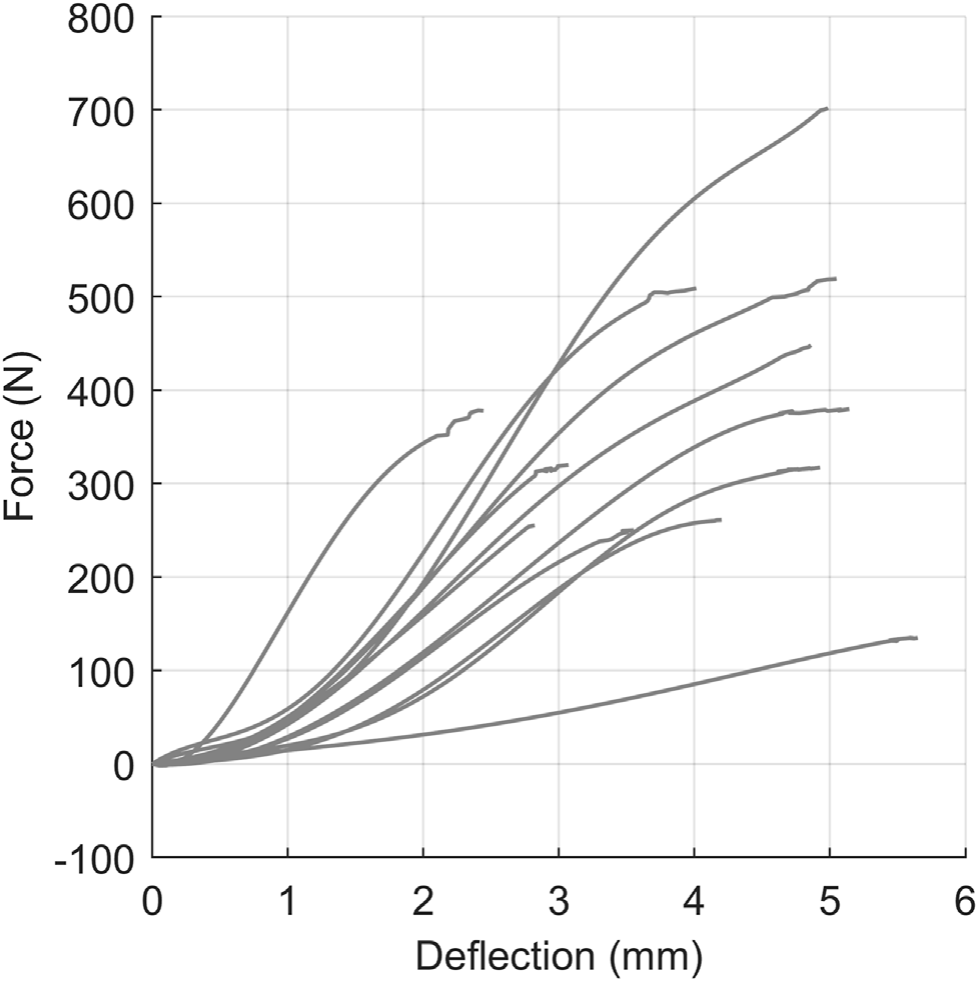
The quasi-static force-displacement response of 12 anterior longitudinal ligament specimens (Mattucci et al., 2012).

Three data sets were assessed using the proposed arc-length corridor method. The first data set, comprising force-displacement data for cervical spine ligaments (Mattucci et al., 2012), demonstrated monotonic behavior with relatively large variability and a lack of shared endpoints. Five ligaments were tested, including the anterior longitudinal ligament (Figure 6), posterior longitudinal ligament, ligamentum flavum, capsular ligament, and interspinous ligament (Supplementary Material S1;Section 1). Force-displacement signals were reproduced from the original experimental data provided by the authors. In keeping with the method used by Mattucci et al., signals were truncated at the end of the traumatic region, excluding post-ultimate load response. Although Mattucci et al. also excluded the traumatic region of some experimental signals because the signals were considered uncharacteristic in shape, no portion of any signal was excluded in the current study. Since the signals were monotonic in nature, no control points were defined ( m=0 ) and therefore signal registration was not applied.

**FIGURE 7:**
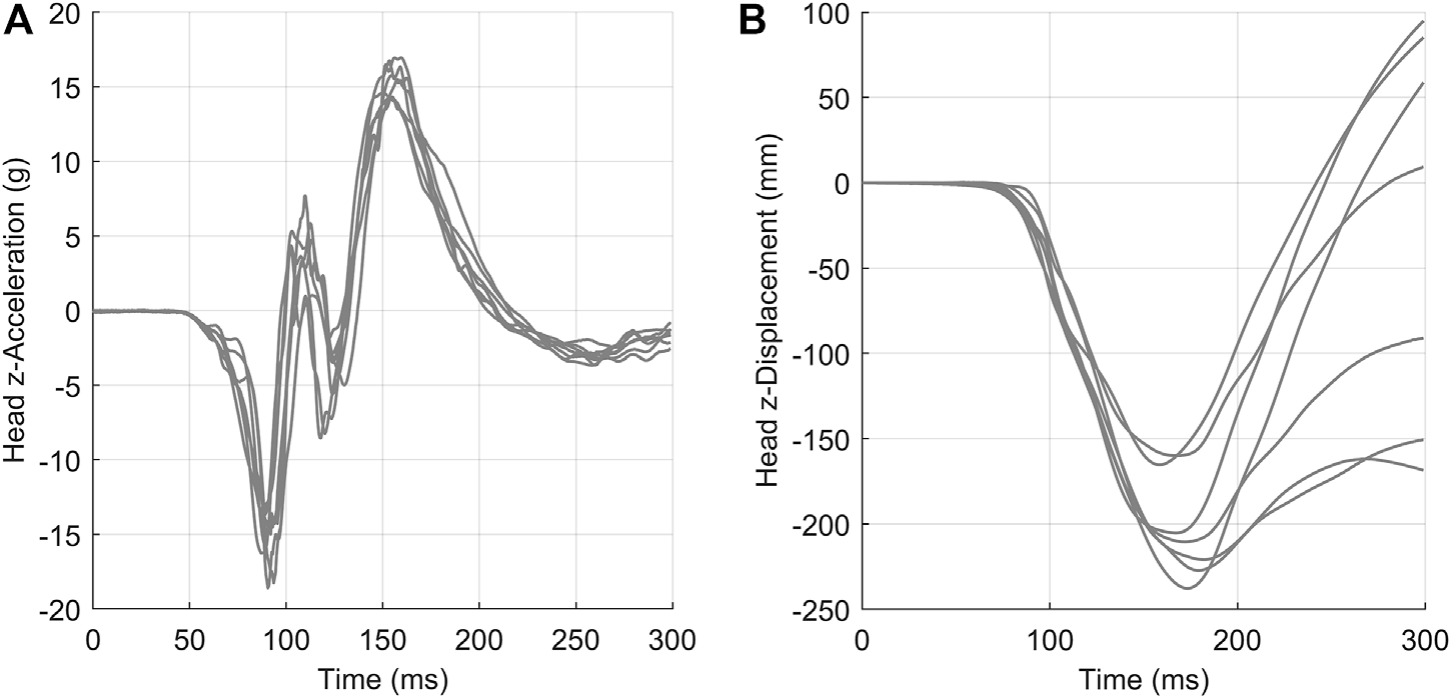
Head acceleration **(A)** and displacement **(B)** in the *z*-direction for a 15 g frontal acceleration. Signals were collected from seven human volunteers.

The second data set, head acceleration data from human volunteer 15 g frontal sled tests (Ewing and Thomas, 1972), was non-monotonic and oscillatory (Figure 7). This study reported head center of gravity kinematics in the global coordinate system, where the x -axis aligned with the acceleration pulse. Experimental acceleration-time and displacement-time signals were retrieved from the National Highway Traffic Safety Administration online database (National Highway Traffic Safety Administration, 2017). Experimental signals were captured at 2000 Hz and published with critical features well aligned. No additional filtering or signal processing was performed prior to applying the arc-length corridor method. Kinematics in the z -axis are highlighted in this paper as they were the most representative of both highly oscillatory (acceleration-time) and potentially divergent (displacement-time) signals. The remaining kinematics are reported in the Supplementary Material S1, Section 2.0.

**FIGURE 8:**
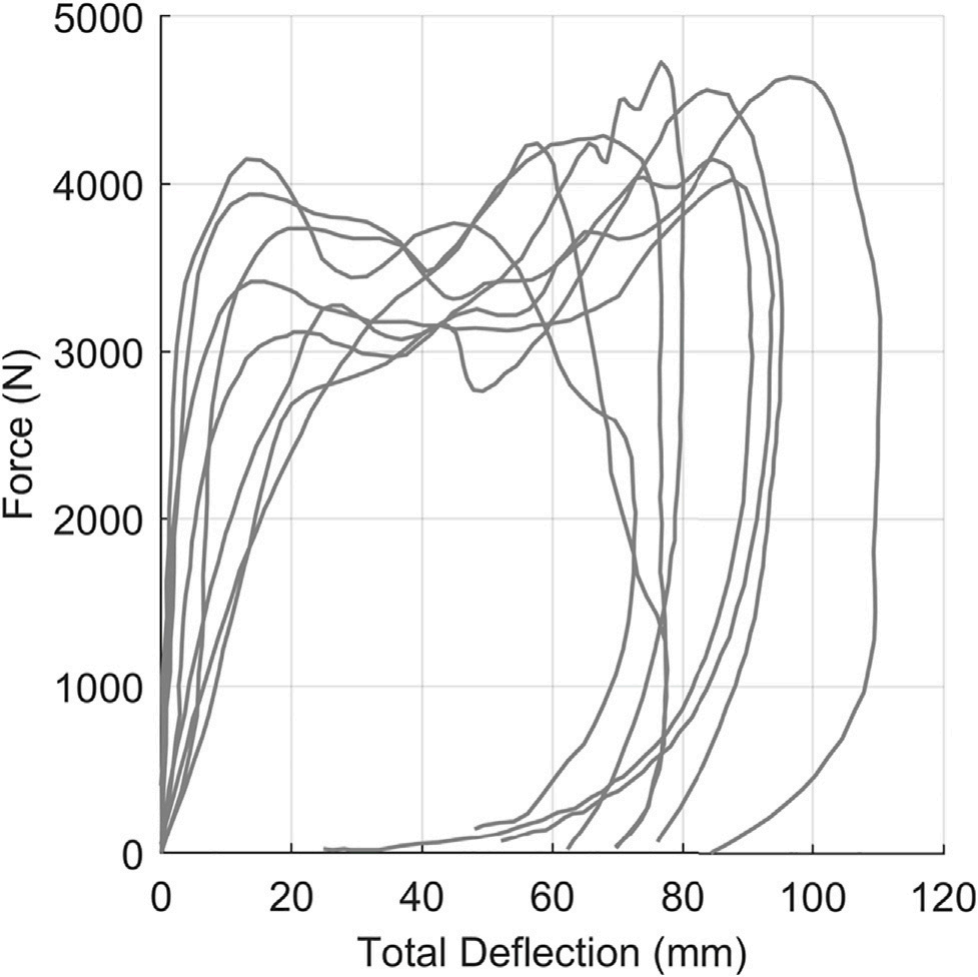
Kroell et al. thoracic impact response signals collected from eight post-mortem human subjects.

The third dataset was force-displacement data from pendulum impacts on the thorax of post-mortem human subjects (Kroell et al., 1971), demonstrating non-monotonic and hysteretic response (Figure 8). Signals used in this work were digitized from the original (Kroell et al., 1971) publication with no additional processing aside from converting measurements to metric units.

## Results

The characteristic average and response corridors were computed for the three literature datasets to demonstrate the efficacy of the arc-length corridor method.

### Dataset 1: Monotonic Cervical Ligament Response Data

**FIGURE 9:**
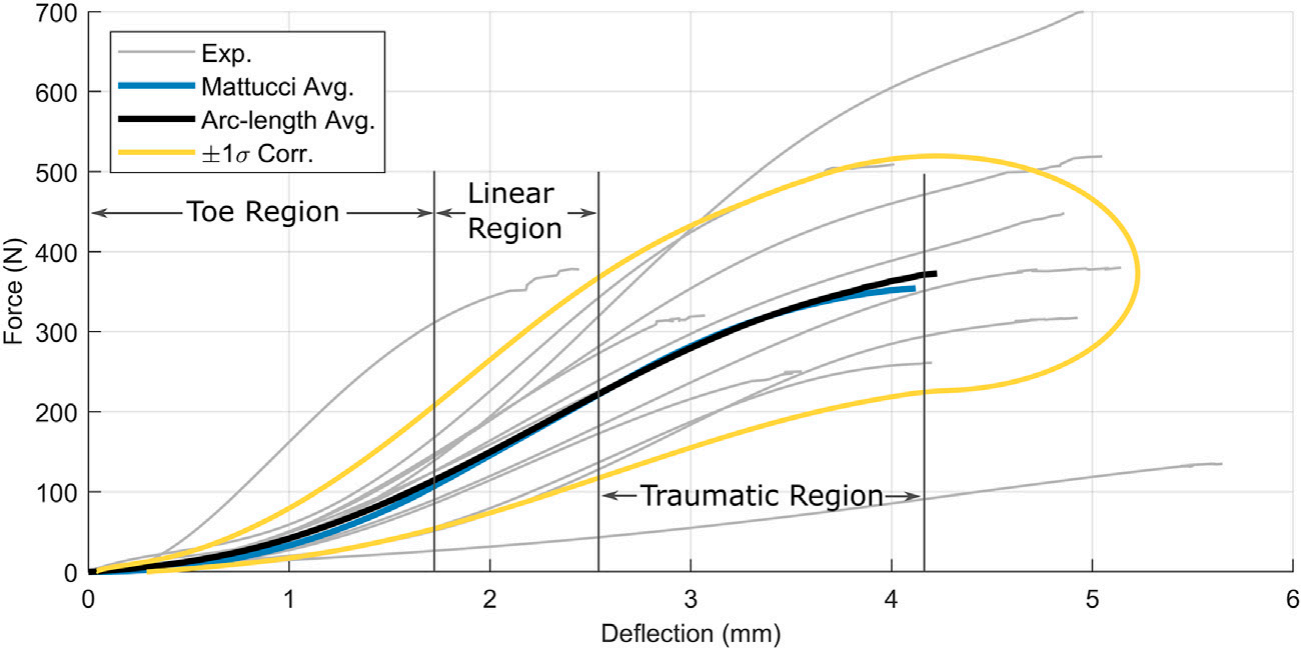
Anterior longitudinal ligament force-displacement responses superimposed with Mattucci average (blue) and arc-length average (black) and ± 1 standard deviation corridors (gold). The three physiological regions of ligament behaviour are labelled: the toe region, the predominantly linear region, and the concave-down traumatic region.

The arc-length corridor method was applied to five cervical ligament quasi-static experimental datasets (Mattucci and Cronin, 2015). Only the results of the Anterior Longitudinal ligament are documented here (Figure 9), with the remainder presented in the Supplemental Material S1;Supplementary Figures S1–5.

In all cases, the arc-length corridor method produced a characteristic average that captured the three physiologic regions of ligament behavior: a toe region, a predominantly linear region, and a concave-down traumatic region. The characteristic average curve from the arc-length method agreed well with that calculated by Mattucci and Cronin (2015). The corridors computed by the arc-length method were defined as ±1 one standard deviation, and demonstrated broadening width with increased displacement.

### Dataset 2: Oscillatory Head Kinematics Data

**FIGURE 10:**
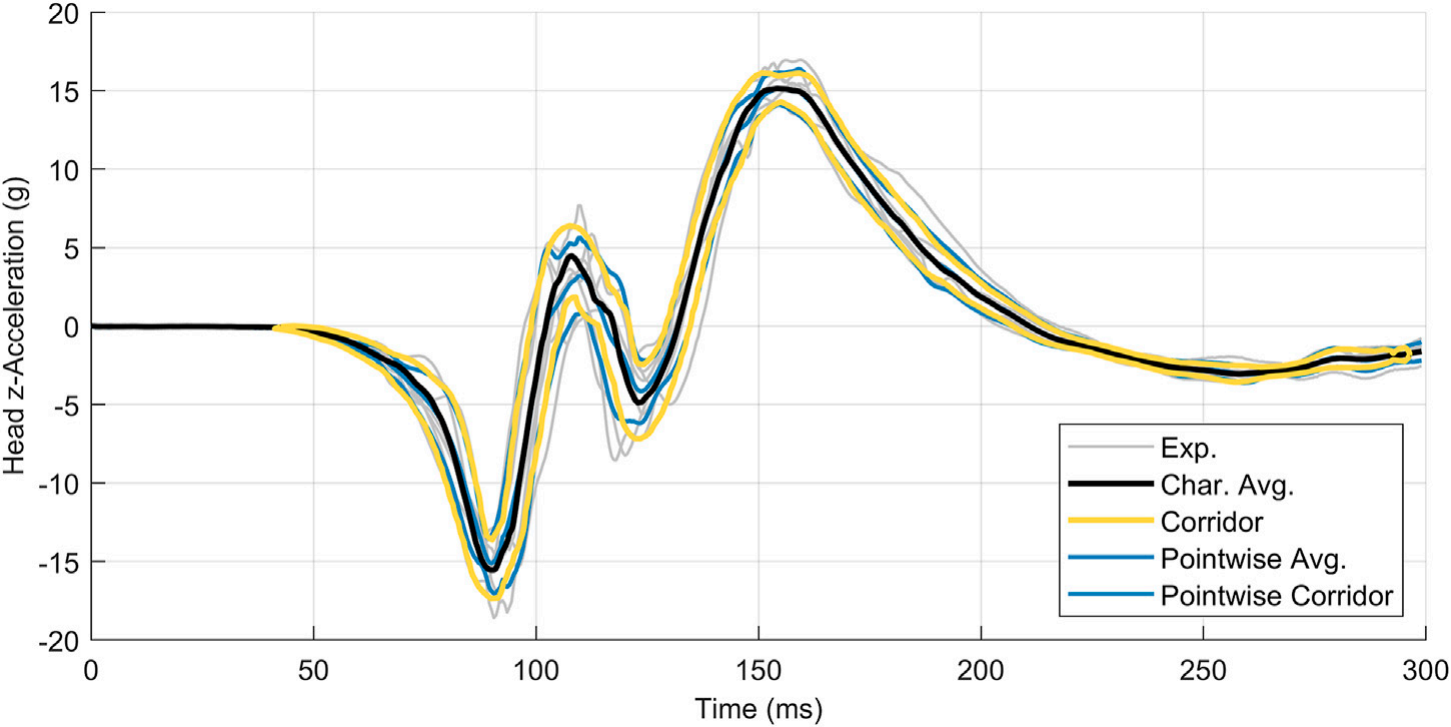
Head z-acceleration for seven subjects, with the characteristic average curve (black) compared to the point-wise average (blue). Corridors are ± 1 standard deviation, m=4 control points, $\lambda =10^{-2}$ .

**FIGURE 11:**
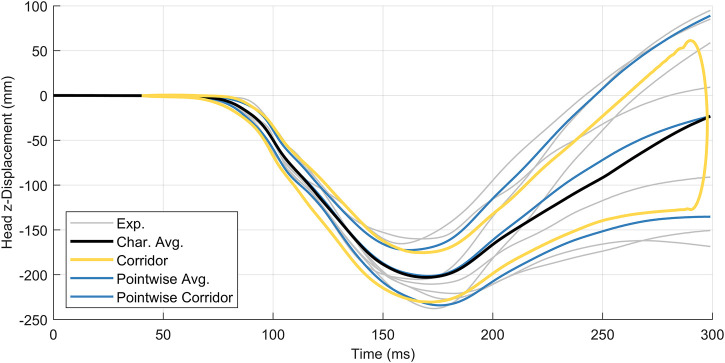
Head z-displacement for seven subjects, with the characteristic average curve (black) compared to the point-wise average (blue). Corridors are ± 1 standard deviation, m=2 control points, $\lambda =10^{-2}$ .

The arc-length corridor method was applied to compute the characteristic average and response corridors for head acceleration (Figure 10) and displacement (Figure 11) in the z -direction (Ewing and Thomas, 1972). The characteristic average and corridors for head kinematics in the *x*-direction and rotation about the y -axis are provided in the Supplementary Figures S6–11. For all load cases, a penalty factor of 10^−2^ was used during signal registration. Four warping control points were used to register the acceleration signals in the *z*-direction while two control points were used for displacement signals. A full accounting of warping control points is provided in Supplementary Table S1 for all kinematics. The calculated corridors were ±1 standard deviation from the average curve. Traditional time-based point-wise average curves and corridors were calculated and presented for comparison.

### Dataset 3: Hysteretic Thoracic Impact Response Data

**FIGURE 12:**
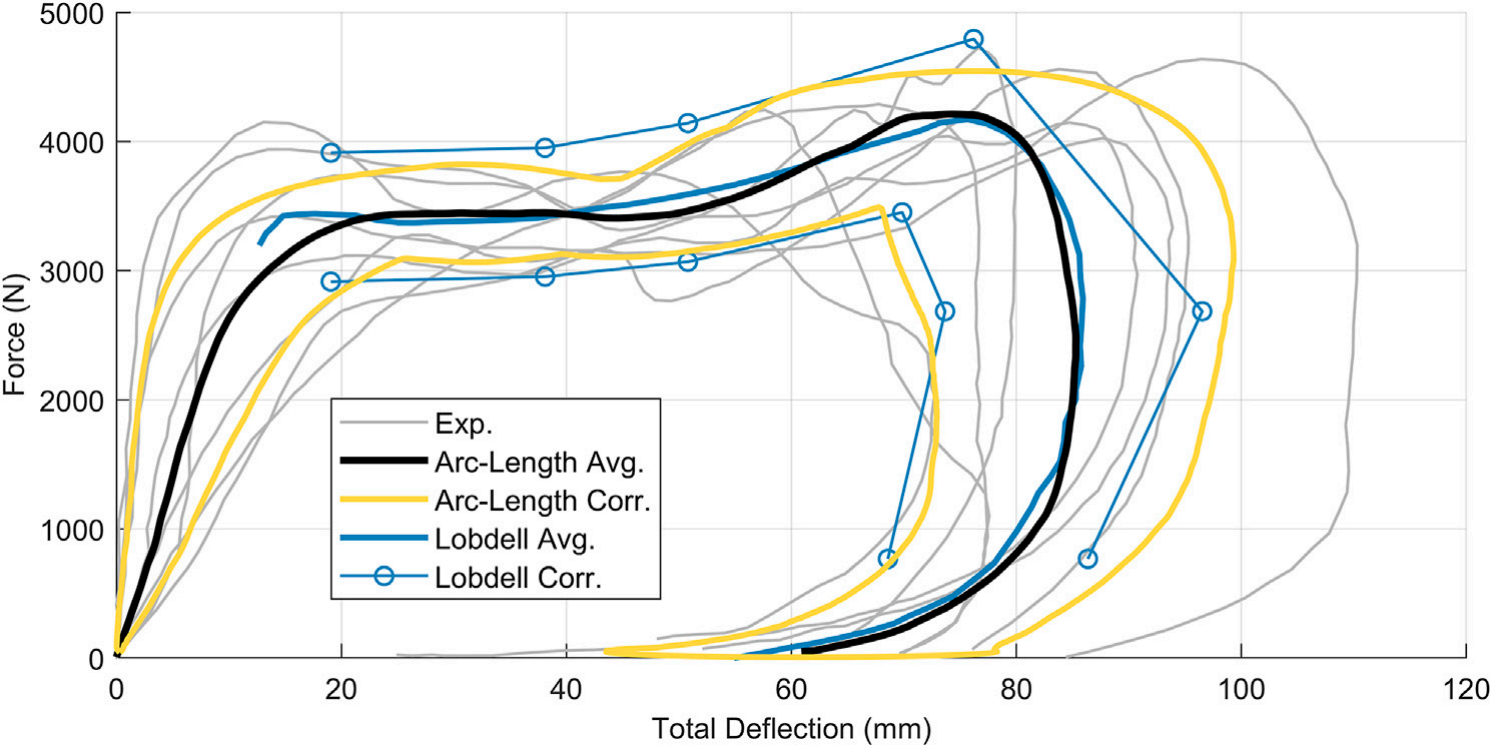
Kroell force-displacement signals overlaid with arc-length average (black) and corridors (gold) as well as the Lobdell corridors (blue). Arc-length corridors are ±1 standard deviation, m = 2 control points, $\lambda =10^{-2}$ .

Application of the arc-length corridor method to the Kroell dataset (Kroell et al., 1971) used two control points ( m=2 ), corresponding to the prominent inflection point seen in the load component and plateau region of the signals (Figure 12). The signal registration penalty factor was $\lambda =10^{-2}$ , and the calculated response corridors were ±1 standard deviation. It should be noted that the arc-length corridor method was applied uniformly to the data, and did not require segmentation as previously documented methods (Lessley et al., 2004) to handle the non-monotonic and hysteretic nature of the signals.

## Discussion

A consistent and reproducible method is needed to determine an average characteristic curve and quantify variability in biomechanical data that may be used as input for computational human models or to assess the output and biofidelity of a model. Such curves are used as input for tissue material properties (Mattucci and Cronin, 2015) in detailed human body models, and can be used to assess the biofidelity of human computational models using methods such as cross-correlation (Barker and Cronin, 2021). Contemporary data averaging and corridor methods are often tailored to a specific data set and require some judgement in application to other signals, such that no general, consistent methodology is available to address the wide variety of data observed in biological tests.

In a review of published methods of computing response corridors, Kim et al. (2013) determined that while most time- and cross-variable-based methods produce broadly similar characteristic averages, the computed response corridors differ to a large extent, likely owing to differences in formulation between each method. Kim et al. judged that a relatively simple time-based, point-wise averaging method produced the best corridors. However, it is important that Kim et al. used a reasonably simplistic synthetic dataset that did not include features unavoidable in experimental data such as noise, non-monotonic behaviour, or signal misalignment. The arc-length corridor method proposed in the current study employs arc-length re-parameterization to enable calculations for non-monotonic data, including load-unload type data without manual signal segmentation. Although differences in magnitude between the axes can skew arc-length calculations and misrepresent features, scaling of signals to compute arc-length minimized distortion. Such differences in magnitude are common in experimental data like force-displacement or stress-strain curves.

The integration of signal registration through the use of warping functions allowed for the automatic alignment of critical signal features (such as peaks and valleys) based on a user-defined number of control points and warping penalty factor. Signal registration addresses a limitation in other arc-length parameterization and point-wise averaging methods, which assume critical features occur at approximately the same value of the dependent variable in all signals. This is a valid assumption for signals that are monotonic in both axes or highly correlated signals that exhibit very little variability. However, this assumption is violated when input signals exhibit variability or may not share all the same features. Further, the utilization of signal registration allowed the characteristic average and response corridors to be computed without smearing or distorting the underlying shape of the data.

The proposed arc-length corridor method was used to analyze three datasets with distinctly varying signal characteristics, a generalization not possible with current methodologies. Monotonic force-displacement human ligament data (Mattucci and Cronin, 2015) demonstrating variability and lack of shared signal endpoints could not be assessed using point-wise averaging. Mattucci and Cronin (2015) proposed a tailored method based on the assumed shape of ligament behaviour. The data was analyzed by segmenting each signal into three characteristic shapes or regions, then normalizing and averaging curves within each region. One consequence of this method was an enforced stationary point at the end of the traumatic region. While this is a valid assumption from a physiologic perspective, such a trait was not readily apparent in the experimental signals.

In contrast, the arc-length corridor method did not contain a priori assumptions of signal shape. The method generated similar average curves to the Mattucci method, which is to be expected since the aim of the Mattucci method was to generate representative average curves tailored to the data set. However, the average curves did differ between methods for the interspinous (Supplementary Figure S5) and Ligamentum Flavum (Supplementary Figure S3). The arc-length corridor method resulted in lower average curves for the traumatic region of these two ligaments. The higher average produced by the Mattucci method may be the result of the assumed curve shape and magnitude difference for a small number of curves in each dataset. Importantly, the arc-length corridor method enabled the generation of statistical corridors, which was not possible using the method developed by Mattucci.

**FIGURE 13:**
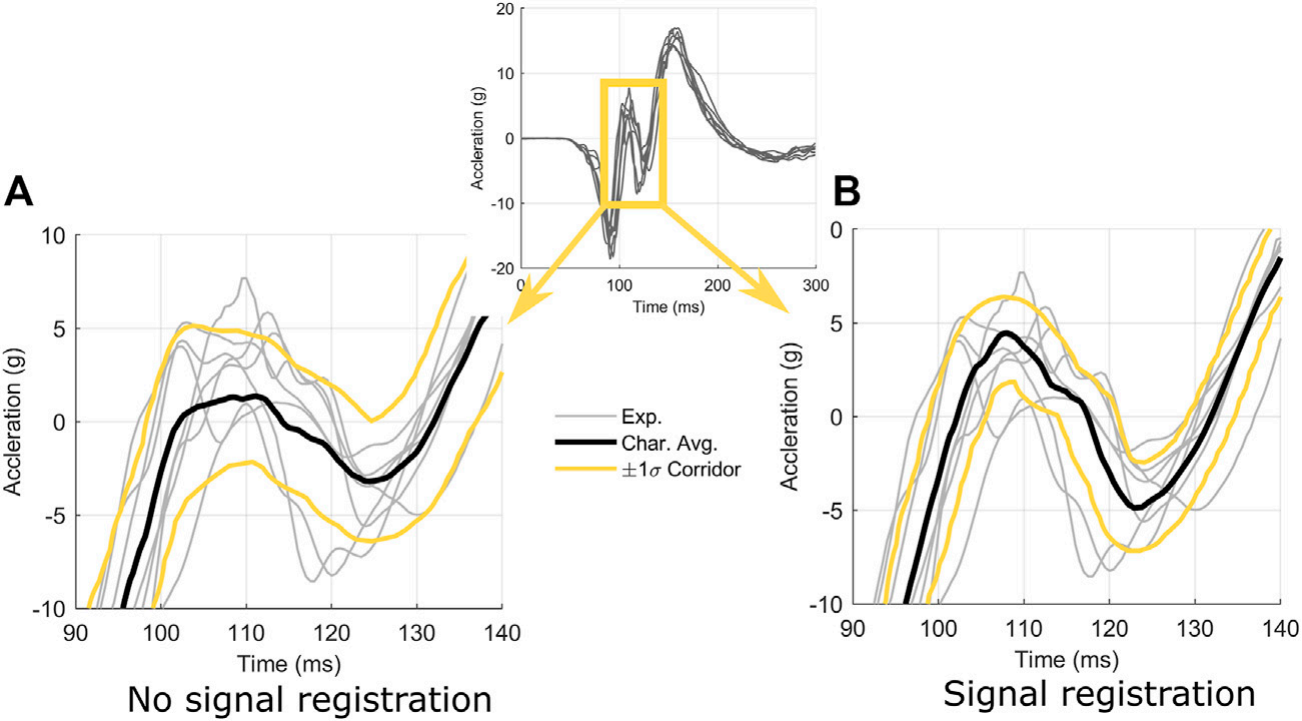
Effect of signal registration on the characteristic average and response corridors for head acceleration in the *z*-direction. Signal registration **(B)** utilized m=4 control points and produced a characteristic average that captured the underlying shape of the signals better than not performing signal registration **(A)**.

The head center of gravity data in the human volunteer sled data (Ewing and Thomas, 1972) were oscillatory in nature and demonstrated low variability. Applying arc-length re-parametrization without signal registration for the head z-acceleration, it was noted that the average and corridors did not capture the true mean height or location of the intermediate peaks centred at a time of approximately 115 m (Figure 13). These peaks were smeared out somewhat due to variations in arc-length between signals resulting from noise and experimental variability (Figure 13A). After performing signal registration with m=4 control points and a penalty factor of $10^{-2}$ , the characteristic average has a more defined peak and larger amplitude between adjacent features that reflect the shape of individual signals better without distortion, while the response corridors conformed better to the shape of the experimental data (Figure 13B). The effect was subtle in this case, owing to the relatively small misalignment between peaks and valleys.

It was noted that the experimental signals exhibited divergent behaviour later in time (Figure 11) made signal registration difficult, where the differences in curve length and diminished cross-correlation between signals. Conversely, the arc-length corridor method produced characteristic averages and corridors that reflected experimental behaviour well in cases where signals were better correlated.

The low variability of the Ewing and Thomas dataset enabled point-wise methods (e.g., Barker and Cronin (2021)) to represent the average response and corridors. The arc-length corridor method produced broadly similar results compared with the time-based point-wise average and corridors computed for each set of signals. However, it is important to consider that this dataset is ideal for using a time-based point-wise average, as all signals were sampled at exactly the same times and published with key features well aligned in time. Not all time-based acceleration data reported in the literature is as well-behaved and highly correlated as the Ewing and Thomas data.

The Lobdell thorax impact response signals are a highly influential dataset in biomechanics, particularly in occupant safety, and serve as the basis of some of the earliest cross-variable response corridors (Lobdell et al., 1972). Of the three datasets examined, the application of the arc-length corridor method to the Kroell et al. thoracic impact dataset presented the largest improvement over other reported techniques. The arc-length corridor method provided the ability to compute a characteristic average and response corridors without segmentation of the signals (Lessley et al., 2004). Furthermore, the statistical underpinning of the corridors from the current methodology provided a significant improvement over the original ± 15% wide corridors presented by Lobdell et al. (1972). The improved resolution of arc-length response corridors also captured how the variability between signals changed throughout the impact event. One interesting feature of the corridors was a constriction around 42 mm of deflection in the plateau region. While it may seem incongruous, this constriction captured the coalescence of input signals around that point.

The arc-length corridor method presents several major advantages over existing techniques documented in the literature as a generalized methodology that can be applied across a wide range of biomechanical signals. While this methodology shares features from the arc-length method documented by Perez-Rapela et al. (2018), it is set apart by a more robust method of re-parameterizing input signals, the incorporation of signal registration to continuously align signal features, and the statistical framework and automated techniques used to extract response corridors. However, the arc-length corridor method does have some limitations. The method relies on the arc-length of a signal, such that high frequency oscillatory signals, such as those arising from noise, could skew results by increasing the computed arc-length of a signal. While signal registration does mitigate this issue to a degree, the arc-length method could produce skewed averages and corridors with extremely oscillatory or noisy input signals. Skewed results can be remedied in one of two ways: filtering the signals (recommended in most cases) or increasing the number of warping control points. In a similar vein, corridors could be skewed should one input signal be significantly longer than the others, such as if one signal in a set of time series responses was collected for twice as long as the others. The simplest solution to this issue is to crop the longest signal; however, cropping should only be applied when physically justified.

While signal registration was shown to be valuable in aligning input signals, especially as seen in the Ewing and Thomas data discussed earlier, signal registration could potentially introduce a degree of subjectivity, as the number of warping control points and penalty factor is somewhat specific to a given dataset. In this study, the number of control points was recommended to be equal to the number of prominent inflection points in the shape of the signal, and this rule of thumb worked well for the data sets considered in the current study. Furthermore, a penalty factor of $\text{λ}=10^{-2}$ was used for all datasets. Unfortunately, there is no general, quantitative method of determining penalty factor value for signal registration (Ramsay and Li, 1998; Ramsay, 2018). A sensitivity study found the data sets in the current study, which all had a relatively high density of data points, were not sensitive to changes in the penalty factor (within an order of magnitude).

**FIGURE 14:**
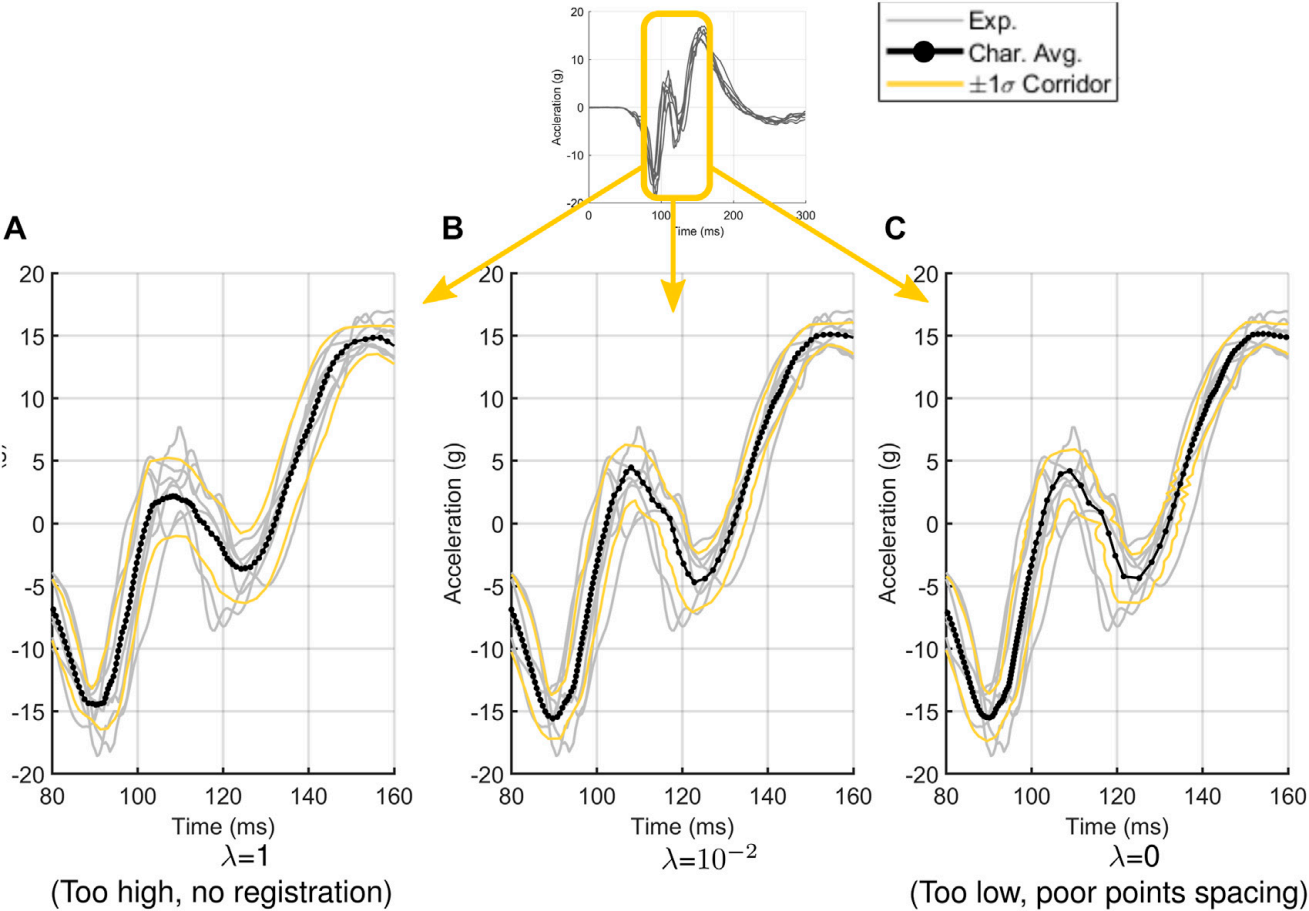
The effect of penalty factor λ on the characteristic average and response corridors for head acceleration in the *z*-direction. If the penalty factor is high **(A)**, signal registration is not performed. If the penalty factor is low **(C)**, signal registration can skew points towards certain features, producing areas of poor discretization and signal resolution. Appropriate penalty factor selection **(B)** produces a curve that captures signal features without significant changes in point density.

Figure 14 demonstrates how varying the penalty factor λ affects the process of signal registration to aid in the selection of this parameter. If λ is set to a high value (e.g., 1.0) (Figure 14A), registration becomes very limited, producing a characteristic average that smears out features like peaks and valleys. This result is effectively the same as not performing registration at all. If λ is small (e.g., 0.0) (Figure 14C), registered points tend to cluster around specific features as the registration process maximizes cross-correlation between signals without regard to the original signal shape. The resulting characteristic average may not capture some critical features or produce poor signal discretization in certain areas, both features that may produce unacceptable response corridors. An appropriate value of λ (Figure 14B) produces a characteristic average that captures critical features without distorting peaks or valleys and without introducing significant changes in signal discretization. While any signal registration will produce a signal where points are not uniformly discretized with respect to arc-length, the appropriate selection of λ will not alter signal point density to a significant degree. To that end, the number of signal points plays some role. For well-discretized signals sampled at a high sampling rate, the changes in density caused by signal registration will have less impact on the resulting average and corridors, making well discretized signals less sensitive to the selection of λ . Poorly discretized signals will be more susceptible to changes in λ as minor changes in signal point density can affect the critical features of a signal to a large degree. In such cases, it is recommended that a range of penalty factor values be evaluated in a parametric study to determine an acceptable value of λ for a given data set. It is highly recommended that researchers report both the number of control points and penalty factor used to produce response corridors to allow for the reproducibility of their results*.*

Finally, while the arc-length corridor technique can accommodate varying sampling rates, the original signal must have a sufficiently high sampling rate to ensure that no distortion is introduced during the re-parameterization and signal registration. Although linear interpolation between discrete signal points was applicable in the current study, if signals were not sampled at a sufficiently high rate, there is a possibility that the resampled signals may not reflect the underlying physics of the original signal. Re-parameterization with higher-order interpolation methods may help prevent distortion of the original signal in these cases. However, such techniques applied to sparsely sampled data present the risk of introducing artifacts if signals are not sufficiently sampled to resolve critical features.

While not a limitation, the arc-length corridor method is agnostic to the physical context of the data. To that end, anthropomorphic scaling, which is commonly applied to data collected from human volunteer or post-mortem human subject trials to account for variation between subject weight, height, and other dimensions, was not performed. Anthropomorphic scaling, such as the equal stress equal velocity method, impulse-momentum approach (Petitjean et al., 2015) or even more advanced techniques (Sun et al., 2016), would generally reduce variability between signals and result in tighter corridors. If required, anthropomorphic scaling can be considered prior to performing corridor generation using the methodology presented in this work. However, care must be taken to ensure that scaling is appropriately applied to avoid the risk of combining data from incongruous subjects. Additionally, while anthropomorphic scaling tends to reduce the variability of the dataset, it is important to note that the dataset no longer represents the subject population, but rather the variability with respect to a specific set of human dimensions.”

## Conclusion

This paper has presented a method of computing the characteristic average and bio-fidelity response corridors based on arc-length re-parameterization. Compared to the existing corridor creation techniques, the method presented in this paper has several advantages. First, re-parameterization of experimental signals allows for the analysis of data that may be hysteretic or may terminate at different locations. Second, variation and misalignment of signal features can be accommodated by using signal registration, preventing distortion or smearing of signal features, a common issue in point-wise averaging methods. Finally, this technique permits the ability to compute and interpret response corridors within a statistical framework when assessing the variability of a dataset.

Overall, the generalized nature of the proposed method allowed for consistent, statistically-based, and repeatable analysis of signals across many data types with a single analysis framework.

## Data Availability

The datasets presented in this study, along with the code used to implement the arc-length corridor methodology ca be found at github.com/DCHartlen/ARCGen.

## Nomenclature

C: Correlation score used for signal registration
$c_{ab}$: Cross-correlation of signal a onto signal b
Λ: Penalty function used for signal registration
λ: Penalty factor used to control warping during signal registration
$h_{i}\left(s\right)$: Warping function for signal i
m: Number of warping control points
max(),min(): The maximum and minimum values in a signal component
n: Number of input signals
$s_{i}$: Arc-length of signal i
$\hat{s}$: Normalized arc-length, uniformly sampled and shared by all signals
SD(): Sample standard deviation
$x_{i},\;y_{i}$: Horizontal and vertical components of signal i
$x_{i,j},\;y_{i,j}$: The j ^th^ point of the horizontal and vertical components of signal i
${\hat{x}}_{i},\;{\hat{y}}_{i}$: Scaled horizontal and vertical components of signal i
$\bar{x},\;\bar{y}$: Mean of a signal component
$\chi_{2}^{2}\left(p\right)$: The chi-square distribution with two degrees of freedom
