# Supplementary material for: Arc-Length Re-Parametrization and Signal Registration to Determine a Characteristic Average and Statistical Response Corridors of Biomechanical Data
Source: Front Bioeng Biotechnol. 2022 Mar 24;10:843148. doi: 10.3389/fbioe.2022.843148 (PMC8987728; doi:10.3389/fbioe.2022.843148)
Supplement: Supplementary file 1 [file DataSheet1.PDF]

Supplementary Material

1 Arc-Length Corridor Method Applied to All Monotonic Cervical Ligament Data

All corridors were for the Mattucci & Cronin (2015) dataset were produced using 250 resampling points during arc-length re-parameterization and 300^2 points during corridor extraction. Signal registration was not applied due to the strictly monotonic nature of the data.

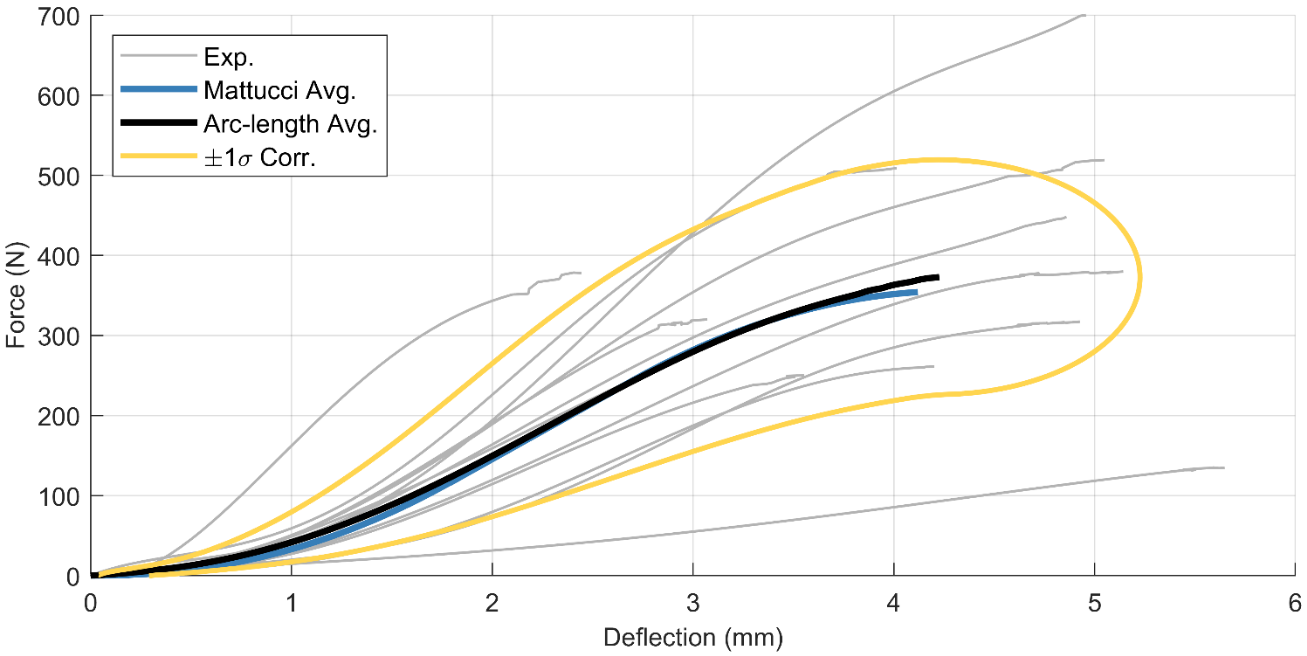

Supplementary Figure 1: Anterior longitudinal ligament force-displacement response superimposed with Mattucci average (blue) and arc-length average (black) and ± 1 standard deviation corridors (gold)

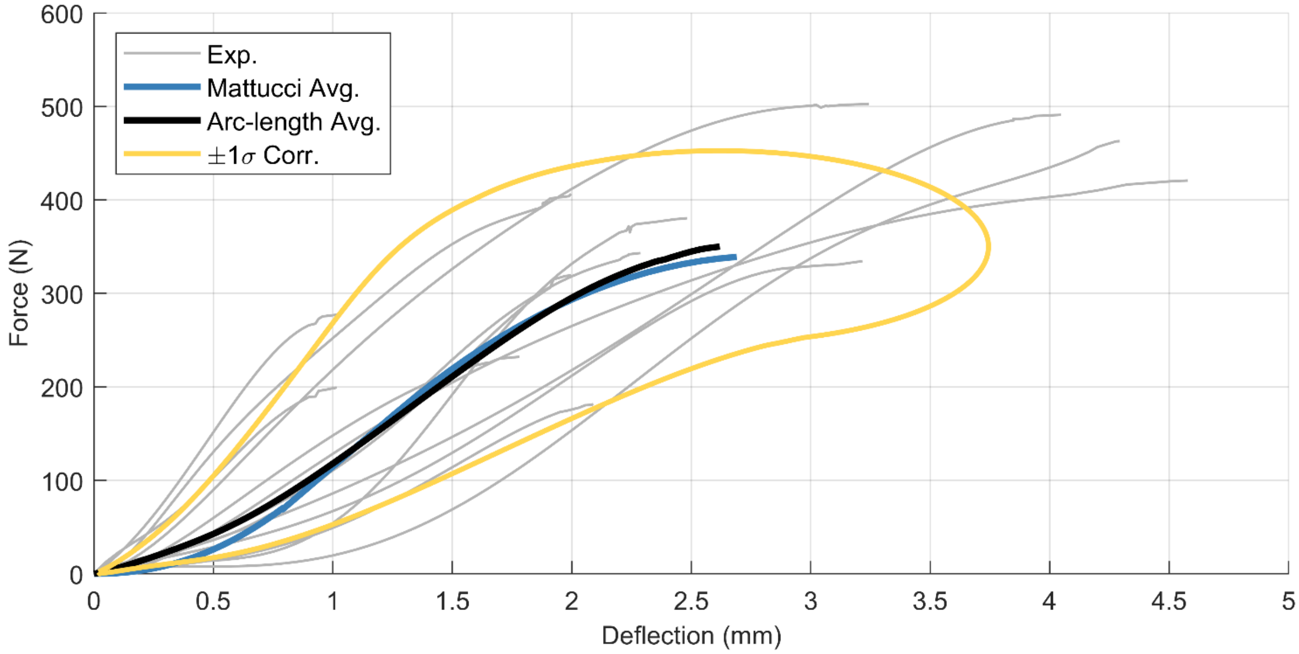

Supplementary Figure 2: Posterior longitudinal ligament force-displacement response superimposed with Mattucci average (blue) and arc-length average (black) and ±1 standard deviation corridors (gold)

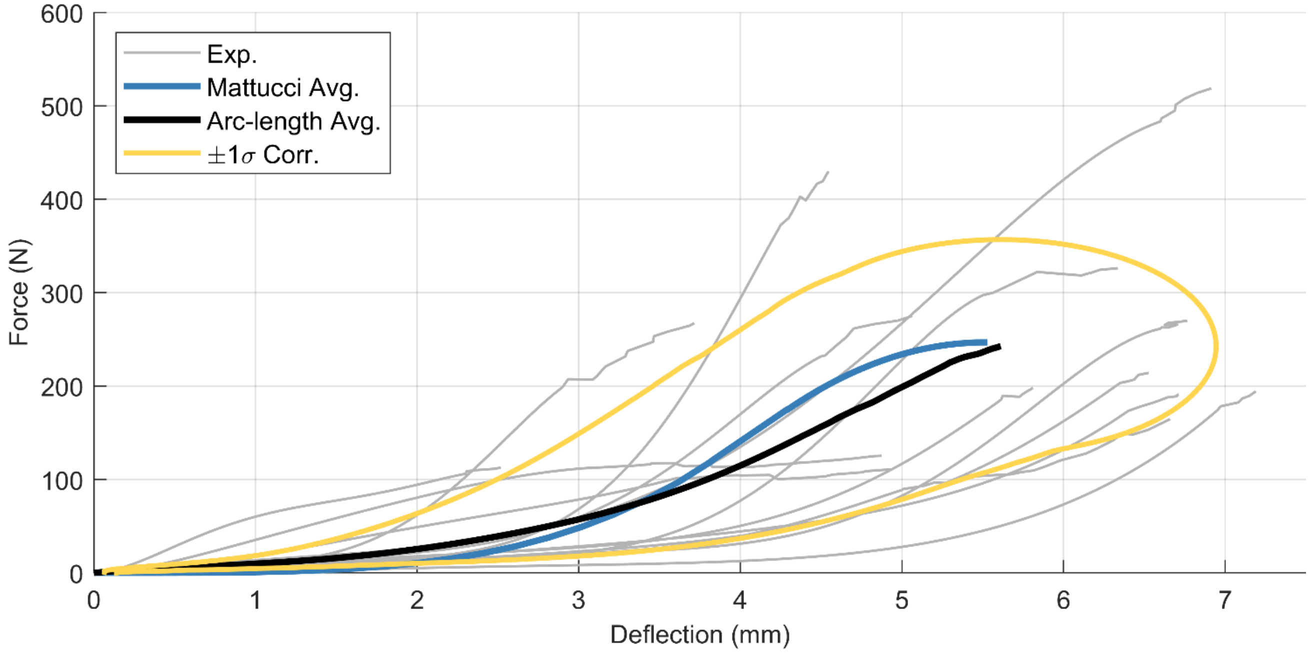

Supplementary Figure 3: Ligamentum flavum force-displacement response superimposed with Mattucci average (blue) and arc-length average (black) and ±1 standard deviation corridors (gold)

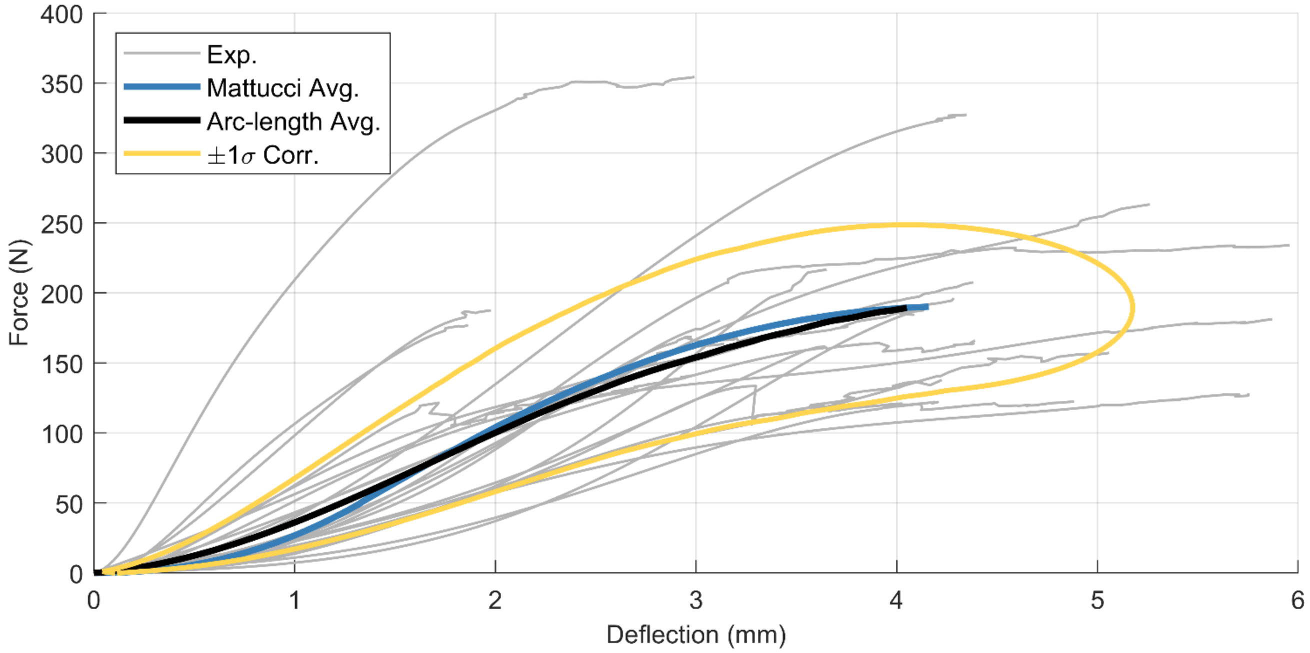

Supplementary Figure 4: Capsular ligament force-displacement response superimposed with Mattucci average (blue) and arc-length average (black) and ±1 standard deviation corridors (gold)

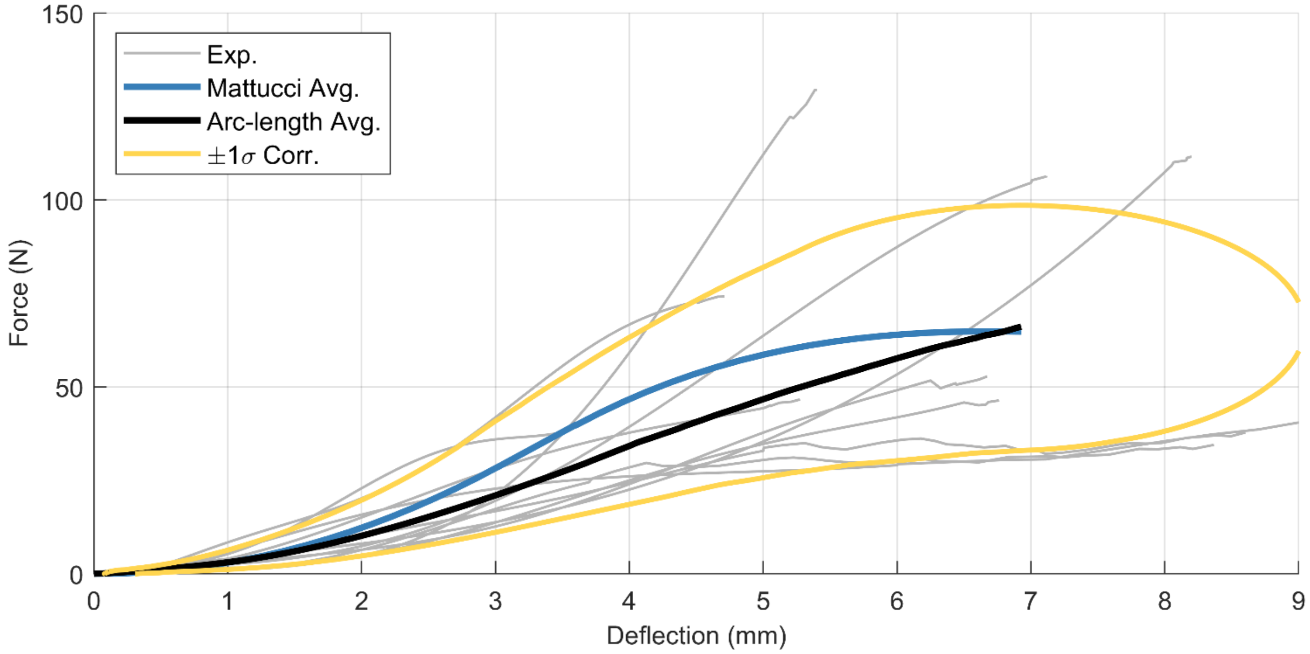

Supplementary Figure 5: Interspinous ligament force-displacement response superimposed with Mattucci average (blue) and arc-length average (black) and ±1 standard deviation corridors (gold)

2 Arc-Length Corridor Method Applied to All Head Kinematics Data

All corridors were for the Ewing & Thomas (1972) dataset were produced using 500 resampling points during arc-length re-parameterization and 500^2 points during corridor extraction. Signal registration was applied to each case. Supplementary Table 1 documents the number of warping control points and penalty factor used for each case.

As all signals were sampled at the same sampling rates and reported at exactly the same times, the point-wise average was computed using time as the independent variable. Corridors for both the arc-length and point-wise methods are ± 1 standard deviation wide. However, while the arc-length method captured uncertainty in both axes, the point-wise method only considered uncertainty in the abscissa.

Supplementary Table 1: Warping control points and penalty factor used for each Ewing & Thomas head kinematic signal set

Case	Warping Control Points	Penalty Factor
x-axis displacement (Supplementary Figure 6)	3	10^{-2}
y-axis rotation (Supplementary Figure 7)	3	10^{-2}
z-axis displacement (Supplementary Figure 8)	2	10^{-2}
x-axis acceleration (Supplementary Figure 9)	4	10^{-2}
y-axis angular acceleration (Supplementary Figure 10)	5	10^{-2}
z-axis acceleration (Supplementary Figure 11)	4	10^{-2}

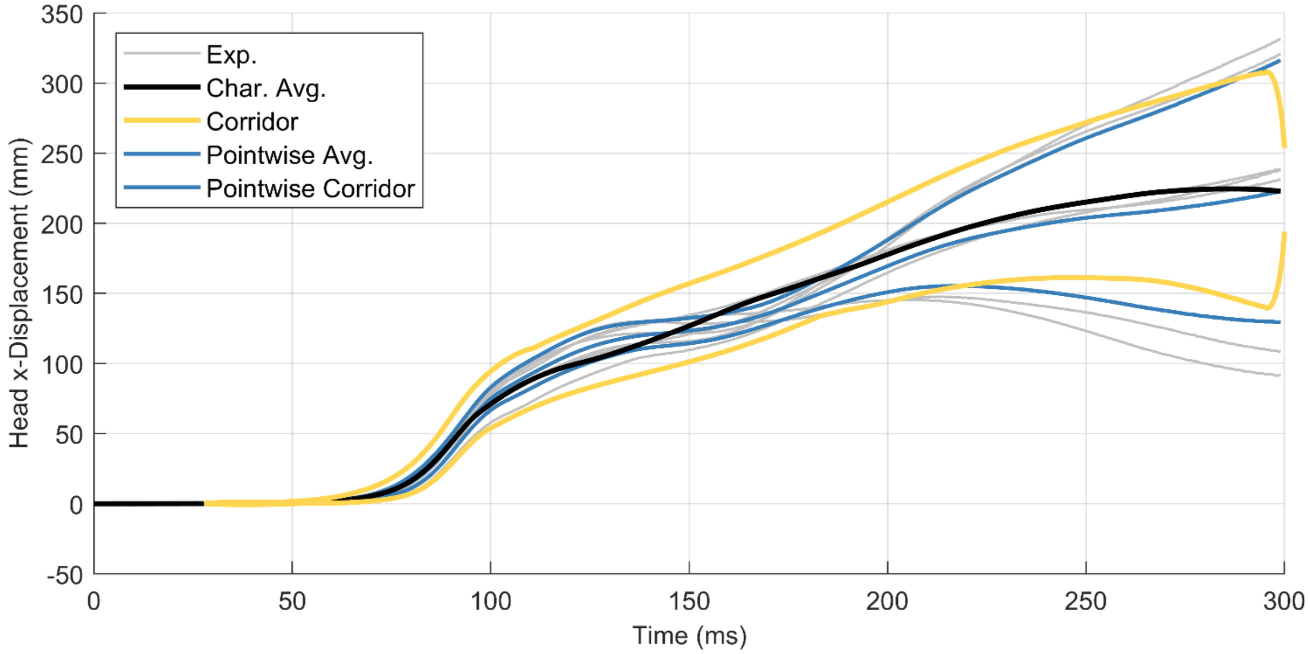

Supplementary Figure 6: Head displacement in the x-direction compared with the point-wise average (blue). Corridors are ± 1 standard deviation, m=3 control points, $\lambda = 10^{-2}$.

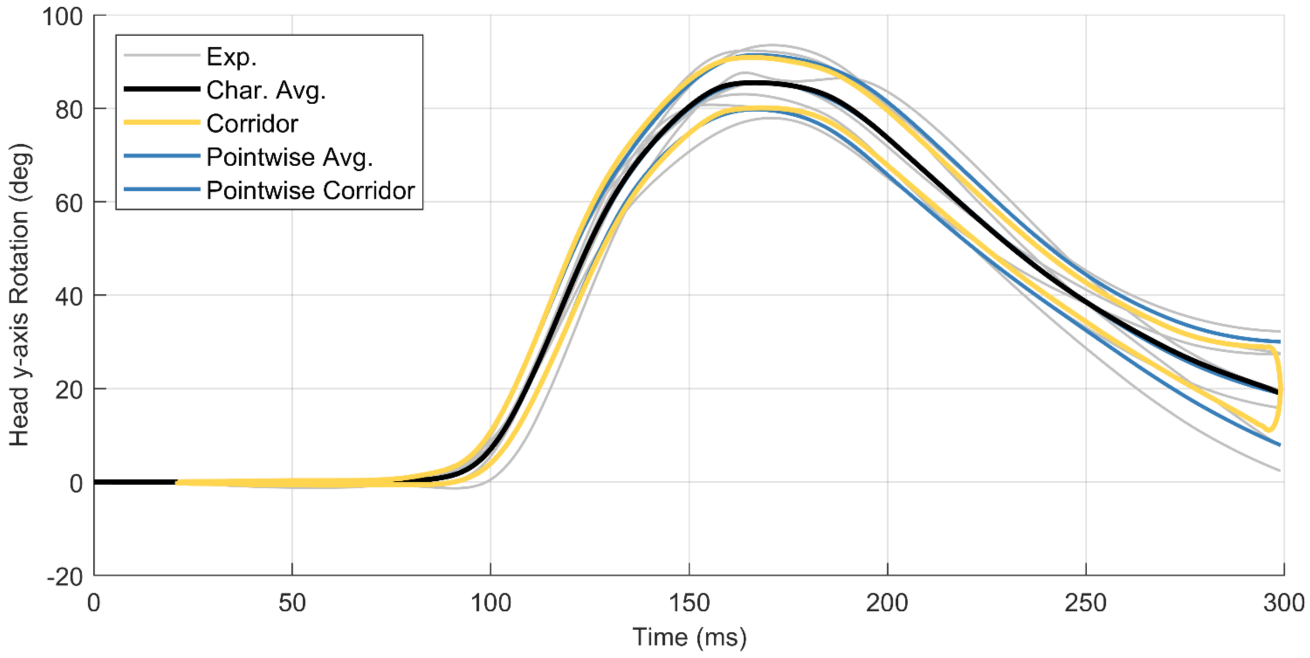

Supplementary Figure 7: Head rotation about the y-axis compared with the point-wise average (blue). Corridors are ± 1 standard deviation, m=3 control points, $\lambda = 10^{-2}$.

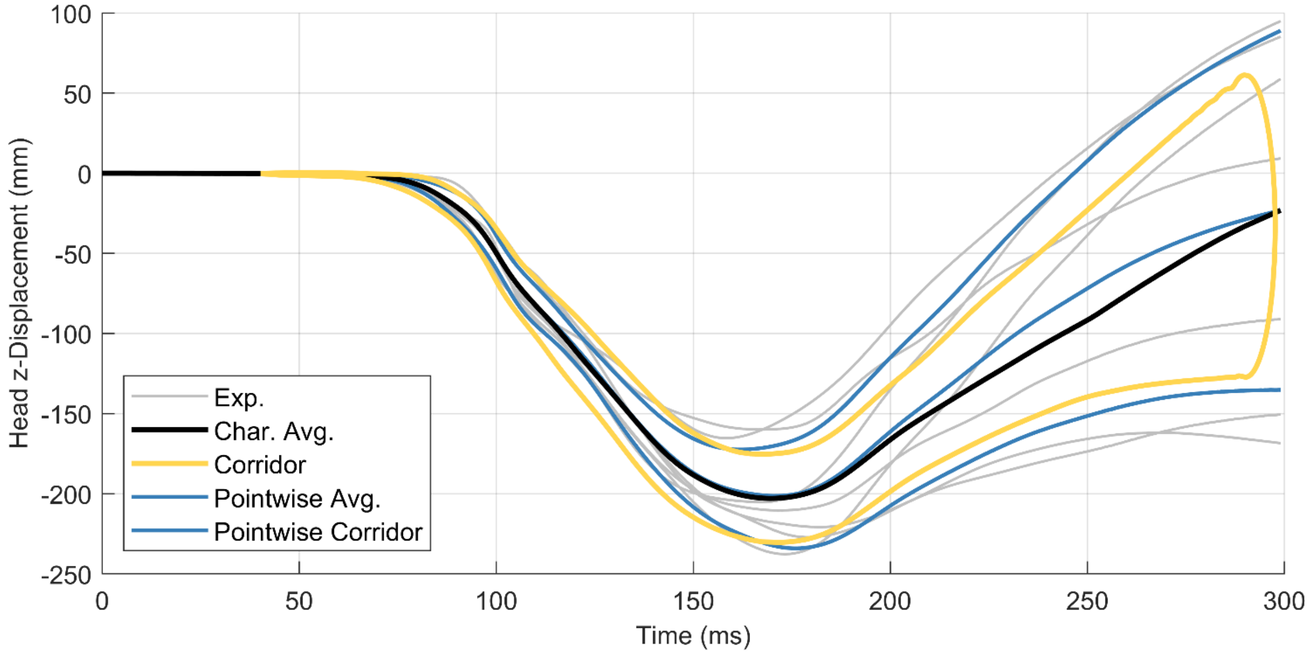

Supplementary Figure 8: Head rotation in the z-direction compared with the point-wise average (blue). Corridors are ± 1 standard deviation, m=3 control points, $\lambda = 10^{-2}$.

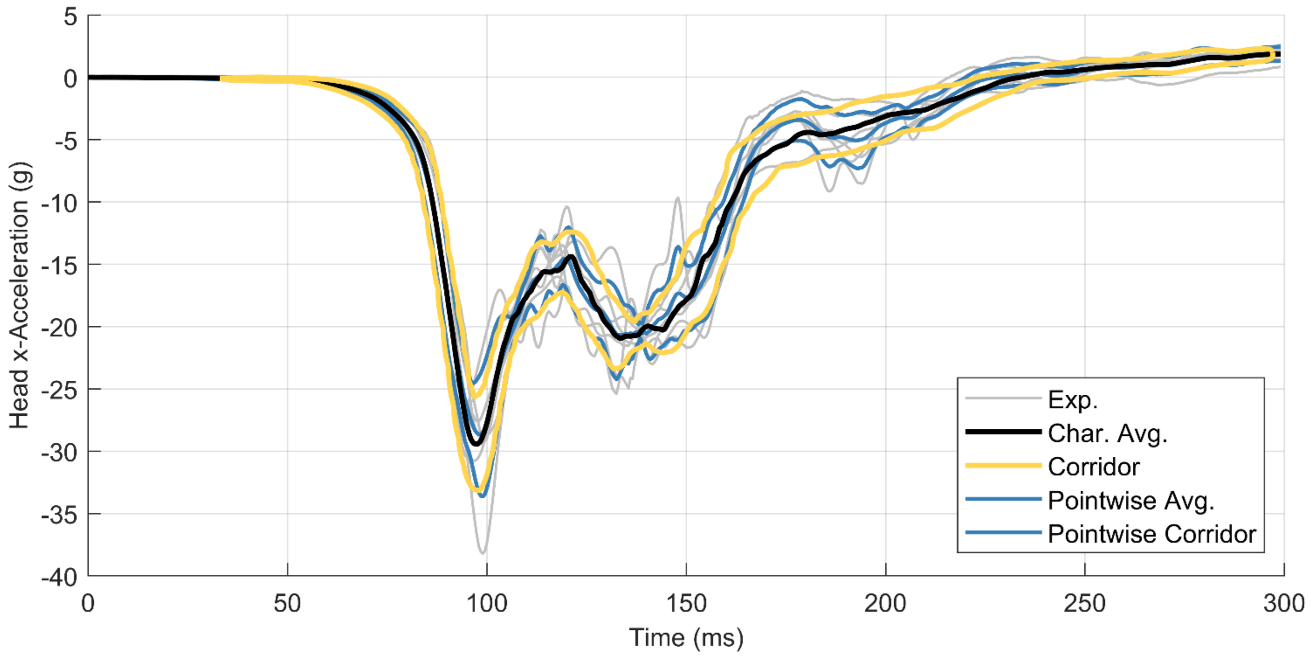

Supplementary Figure 9: Head acceleration in the x-axis compared with the point-wise average (blue). Corridors are ± 1 standard deviation, m=4 control points, $\lambda = 10^{-2}$.

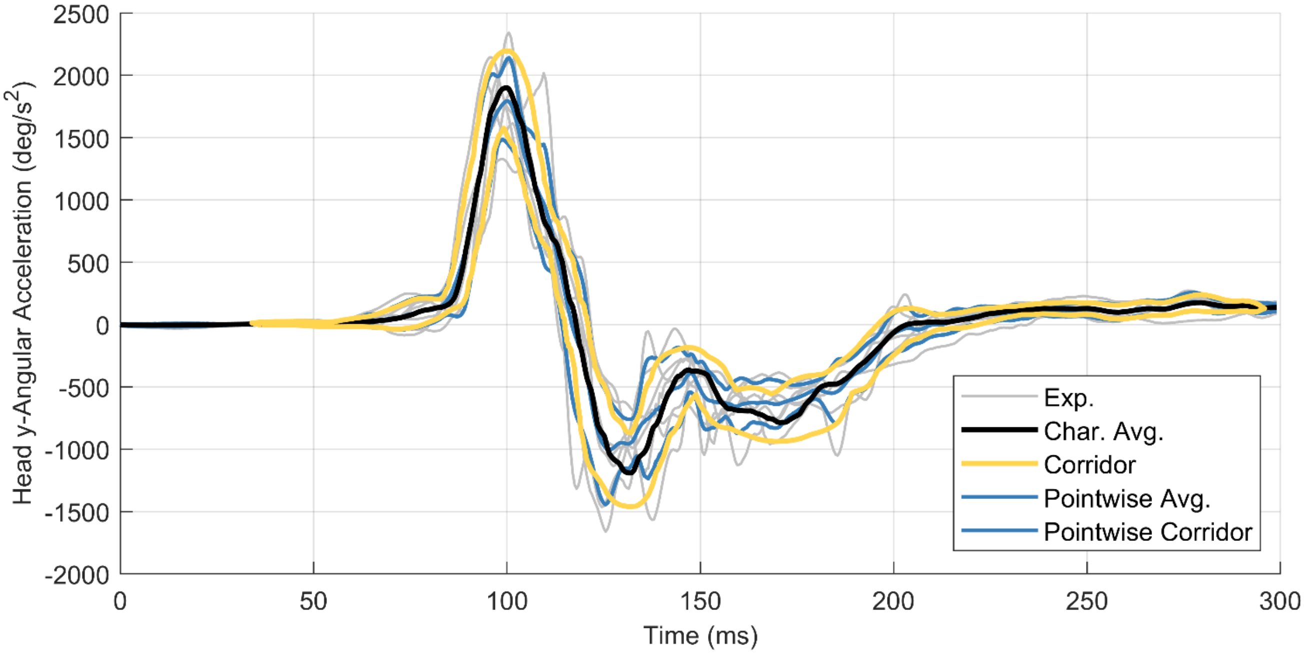

Supplementary Figure 10: Head rotational acceleration about the y-axis compared with the point-wise average (blue). Corridors are ± 1 standard deviation, m=5 control points, $\lambda = 10^{-2}$.

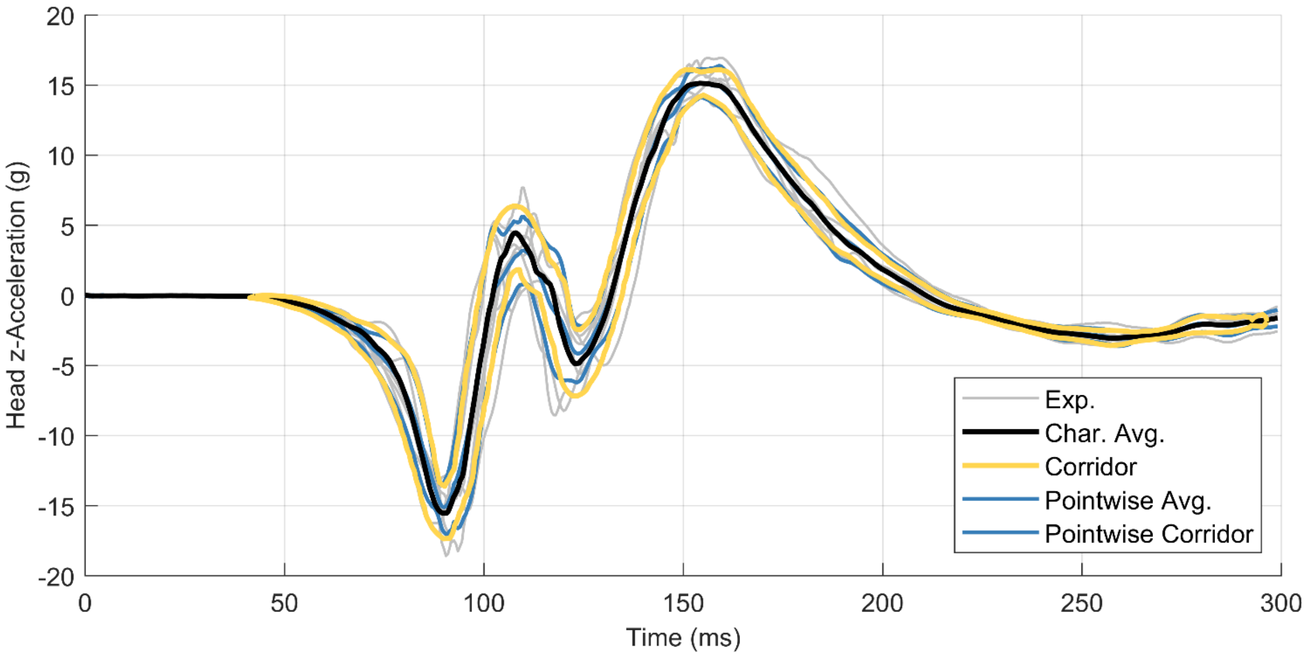

Supplementary Figure 11: Head acceleration in the z-axis compared with the point-wise average (blue). Corridors are ± 1 standard deviation, m=4 control points, $\lambda = 10^{-2}$.
